# Supplementary material for: Patient and practitioner perspectives on the design of a simulated affective touch device to reduce procedural anxiety associated with radiotherapy: a qualitative study
Source: BMJ Open. 2022 Mar 16;12(3):e050288. doi: 10.1136/bmjopen-2021-050288 (PMC8928300; doi:10.1136/bmjopen-2021-050288)
Supplement: Author's manuscript [file bmjopen-2021-050288.draft_revisions.pdf]

BMJ Open is committed to open peer review. As part of this commitment we make the peer review history of every article we publish publicly available.

When an article is published we post the peer reviewers' comments and the authors' responses online. We also post the versions of the paper that were used during peer review. These are the versions that the peer review comments apply to.

The versions of the paper that follow are the versions that were submitted during the peer review process. They are not the versions of record or the final published versions. They should not be cited or distributed as the published version of this manuscript.

BMJ Open is an open access journal and the full, final, typeset and author-corrected version of record of the manuscript is available on our site with no access controls, subscription charges or pay-per-view fees (http://bmjopen.bmj.com).

If you have any questions on BMJ Open's open peer review process please email info.bmjopen@bmj.com

BMJ Open

PATIENT AND PRACTITIONER PERSPECTIVES ON THE DESIGN OF A SIMULATED AFFECTIVE TOUCH DEVICE TO REDUCE PROCEDURAL ANXIETY ASSOCIATED WITH RADIOTHERAPY: A QUALITATIVE STUDY

Journal:	*BMJ Open*
Manuscript ID	bmjopen-2021-050288
Article Type:	Original research
Date Submitted by the Author:	24-Feb-2021

Complete List of Authors:	Hall, Hugo ; King's College London; Imperial College London<br>Dhuga, Yasmin; Brighton and Sussex Medical School; Imperial College London<br>Yan Zheng, Caroline; Royal College of Art<br>Clunie, Gemma; Imperial College London Department of Surgery and Cancer, Sackler MSK Lab; Imperial College Healthcare NHS Trust, Speech and Language Therapy<br>Joyce, Elizabeth; Royal Marsden NHS Foundation Trust<br>McNair, Helen; Institute of Cancer Research, Radiotherapy and Imaging ; Royal Marsden NHS Foundation Trust<br>McGregor, Alison; Imperial College London, Dept of Surgery and Cancer

Keywords:	RADIOTHERAPY, QUALITATIVE RESEARCH, ONCOLOGY

SCHOLARONE™ Manuscripts

BMJ

I, the Submitting Author has the right to grant and does grant on behalf of all authors of the Work (as defined in the below author licence), an exclusive licence and/or a non-exclusive licence for contributions from authors who are: i) UK Crown employees; ii) where BMJ has agreed a CC-BY licence shall apply, and/or iii) in accordance with the terms applicable for US Federal Government officers or employees acting as part of their official duties; on a worldwide, perpetual, irrevocable, royalty-free basis to BMJ Publishing Group Ltd ("BMJ") its licensees and where the relevant Journal is co-owned by BMJ to the co-owners of the Journal, to publish the Work in this journal and any other BMJ products and to exploit all rights, as set out in our licence.

The Submitting Author accepts and understands that any supply made under these terms is made by BMJ to the Submitting Author unless you are acting as an employee on behalf of your employer or a postgraduate student of an affiliated institution which is paying any applicable article publishing charge ("APC") for Open Access articles. Where the Submitting Author wishes to make the Work available on an Open Access basis (and intends to pay the relevant APC), the terms of reuse of such Open Access shall be governed by a Creative Commons licence – details of these licences and which Creative Commons licence will apply to this Work are set out in our licence referred to above.

Other than as permitted in any relevant BMJ Author's Self Archiving Policies, I confirm this Work has not been accepted for publication elsewhere, is not being considered for publication elsewhere and does not duplicate material already published. I confirm all authors consent to publication of this Work and authorise the granting of this licence.

For peer review only

PATIENT AND PRACTITIONER PERSPECTIVES ON THE DESIGN OF A SIMULATED AFFECTIVE TOUCH DEVICE TO REDUCE PROCEDURAL ANXIETY ASSOCIATED WITH RADIOTHERAPY: A QUALITATIVE STUDY

Corresponding/senior authors:

Alison McGregor (a.mcgregor@imperial.ac.uk), Helen McNair (Helen.McNair@rmh.nhs.uk)

Co-authors:

Hugo Hall, Yasmin Dhuga, Caroline Yan Zheng, Gemma Clunie, Elizabeth Joyce, Helen McNair, Alison McGregor.

Hugo Hall and Yasmin Dhuga contributed equally to this paper.

Word count: 3842
Abstract – 226

Funding statement:

This research project was funded by the CRUK Convergence Science Centre at The Institute of Cancer Research, London, and Imperial College London (A26234)

We also acknowledge NHS funding to the NIHR Biomedical Research Centre at The Royal Marsden and The Institute of Cancer Research. The views expressed in this publication are those of the authors and not necessarily those of those of the NHS or the National Institute for Health Research.

Helen McNair is funded by a National Institute for Health Research and Health Education England (HEE/NIHR), Senior Clinical Lecturer award (ICA-SCL-2018-04-ST2-002).

Gemma Clunie is funded by the National Institute for Health Research (NIHR) Imperial Biomedical Research Centre and the NIHR Clinical Doctoral Research Fellowship Programme CDRF-2017-03-028/ Integrated Clinical Academic Programme.

Alison McGregor acknowledges support from the NIHR Imperial Biomedical Research Centre (BRC).

Disclosure Statement: No potential conflict of interest is reported by the authors.

Data Statement: Data (focus group transcripts) is held at Royal Marsden NHS Foundation Trust and can be accessed on request by contacting: Helen.McNair@rmh.nhs.uk

For peer review only

ABSTRACT

Objective The aim of this study was to elicit the views of relevant stakeholders on the design of a device using simulated affective touch to reduce procedural anxiety surrounding radiotherapy and imaging.

Design This qualitative study collected data from focus groups which were then analysed using inductive thematic analysis in line with Braun and Clarke's methods.

Participants and setting Twenty patients and carers were recruited, as well as ten healthcare practitioners involved in either delivering radiotherapy or imaging procedures.

Results Patients, carers and healthcare practitioners agreed on some aspects of the device design, such as ensuring the device is warm and flexible in where it can be used on the body. However, patient and healthcare practitioner cohorts had at times differing viewpoints. For example, healthcare practitioners provided professional perspectives and required easy cleaning of the device. Meanwhile patients focused on anxiety relieving factors, such as the tactile sensation of the device being either a vibration or pulsation. There was no consensus on who should control the device.

Conclusions The desired features of a simulated affective touch device have been investigated. Different priorities of patients, carers and healthcare practitioners were evident. Any design must incorporate such features as to appease both groups. Areas where no consensus was reached could be further explored, alongside including further patient and public involvement in the form of a project advisory group.

Key words: qualitative, focus groups, radiotherapy, procedural anxiety, tactile sensation

For peer review only

ARTICLE SUMMARY

STRENGTHS AND LIMITATIONS OF THIS STUDY

Strength This is the first qualitative study to explore whether a tactile intervention provides a useful tool in reducing procedural anxiety for radiotherapy patients.

Strength This study explores the opinions of two separate stakeholder cohorts, each with different viewpoints (personal and professional) around the design of the device.

Limitation All participants were selected from a single geographical area which may indicate a homogenous focus group composition.

Limitation Our study cannot be generalised to all individuals who receive radiotherapy, as this study only considered adult participants.

For peer review only

For peer review only - http://bmjopen.bmj.com/site/about/guidelines.xhtml

PATIENT AND PRACTITIONER PERSPECTIVES ON THE DESIGN OF A SIMULATED AFFECTIVE TOUCH DEVICE TO REDUCE PROCEDURAL ANXIETY ASSOCIATED WITH RADIOTHERAPY: A QUALITATIVE STUDY

INTRODUCTION

In the UK 458,000 people are currently living with cancer and this is expected to rise to 596,000 people by 2040 (1). Over 50% of these patients will experience radiotherapy as part of their management (2, 3). Radiotherapy commonly causes procedural anxiety with up to 49% of patients experiencing anxiety and psychological distress (4, 5). This problem is likely to be exacerbated with new developments in radiotherapy, for example stereotactic and adaptive radiotherapy, requiring increased time in the treatment room.

High levels of distress during radiotherapy can directly impact the accuracy and efficacy of the procedure (6). However, the consequences of procedural anxiety extend beyond radiotherapy and include diagnostic imaging, and other invasive procedures performed on the conscious patient. Between 2-5% of magnetic resonance imaging (MRI) scans are terminated due to procedural anxiety (7, 8); this equates to a significant financial cost for the NHS considering that 3.4 million MRI scans were conducted between 2017 to 2018 (9).

Pre-existing interventions to manage procedural anxiety are imperfect and there is a wider need for an appropriate anxiety alleviating strategy or device in the medical setting (10-12). Techniques such as verbal communication or music may distract the patient but do not address the reported sense of isolation associated with separation from caregivers or families. Sedation is commonly used to tackle procedural anxiety in the context of MRI but has implications for cost, risk and time (8).

Tactile interventions have not been widely explored in the literature as a tool to reduce procedural anxiety in radiotherapy patients. Simulated affective touch (SAT) interventions has been shown to both relieve psychological distress (13) and crucially, reduce the sense of social isolation associated with being apart from caregivers (14-16).

The following paper aims to investigate both patient and healthcare professional (HCP) views on the creation of a calming device using SAT to combat induced procedural anxiety. Patients can advise on what aspects of any SAT device they would find calming and collecting the views of HCPs as key stakeholders in any potential device, is important to ensuring the device is put into use and does not interfere with their challenging workload.

For peer review only - http://bmjopen.bmj.com/site/about/guidelines.xhtml

METHODS

Study Design

This was a qualitative co-design study using focus groups to elicit the views of two distinct categories of participants, patients and healthcare professionals, on the creation of an SAT device for use during radiotherapy interventions.

We adopted a phenomenological approach using inductive thematic analysis. This allowed us to focus on how best to use the product design to counter the phenomena of procedural anxiety around radiotherapy and imaging.

The study protocol was approved by Greater Manchester South Research Ethics Committee, and permission to conduct the research was granted by Health and Care Research Wales.

Participants and Setting

Participants were recruited from two groups of stakeholders: patients, carers and health care professionals. Patients and carers were invited to participate as experts on the lived experience of having radiotherapy and/or imaging procedures; this allowed us to explore circumstances and situations that may have created anxiety and to provide ideas on how to manage these. The views of healthcare practitioners (HCPs) involved in delivering radiotherapy or imaging procedures were important as key stakeholders if the device is to become part of clinical practice.

Purposive sampling was used to select both patient groups. Inclusion criteria stipulated participants were aged over 18 years, while those patients who were considered too unwell (by self-report or in consultation with clinical staff) were excluded. Those who were unable to understand English without an interpreter were also excluded. Patients and their carers were contacted using waiting room flyers while HCPs were approached by departmental email. Information sheets were then distributed to interested participants who then gave informed consent to join the study.

Data Collection

A total of six focus groups took place at the Royal Marsden Hospital. The total number of participants was 30, with four patient and caregiver groups and two HCP groups. Recruitment was stopped after 6 focus groups when initial analysis of the themes demonstrated no new emerging data, indicative of saturation (17).

For peer review only

	Patient had experience of radiotherapy	Cared for someone with radiotherapy experience	Other	Worked as a healthcare practitioner in radiotherapy
**Totals**	13	3	4	10
**Gender**				
**Male**	4	1	3	-
**Female**	9	2	1	10
**Age**				
**18-25**	-	-		1
**26-35**	-	-		5
**36-45**	-	-		2
**46-55**	2	-		2
**56-65**	7	1	2	-
**66-75**	3	2	2	-
**76-85**	1	-		-

Focus groups were recorded and transcribed verbatim for analysis. Three qualitative researchers of mixed experience and a design researcher were responsible for facilitating the focus groups. All 3 qualitative researchers had no prior relationship with the patient participants but two of them were well known to the HCP cohort. However, the varied comments and depth of discussion implies that that there was freedom for articulating thoughts. In addition, the design researcher was not known to any participants.

Data Analysis

Thematic analysis was used to identify themes and sub-themes inductively within the focus group data. In line with Braun and Clarke's (18) methods, the initial step was for researchers to familiarise themselves with the interview recordings. Next, transcripts were analysed using QSR NVivo Release 1.1 and all information relating to calming measures was coded. Each transcript was double coded (once by the design researcher and once by the medical researchers) allowing for multiple perspectives on the analysis. An independent researcher then reviewed the codes to increase inter-coder reliability and the trustworthiness of the results (19). Periodic meetings about the analysis with the whole project team led to iterations and finally the collation of the codes into themes and sub-themes.

For peer review only - http://bmjopen.bmj.com/site/about/guidelines.xhtml

Patient and Public Involvement

The concept was presented at initial planning stages to two Royal Marsden NHS patient and public groups. Positive feedback was received which enabled us to proceed with study.

RESULTS

Three overarching themes emerged from analysis of the data: patient views on what they want from the device, HCP views on the device and HCP views on what they believe patients want from the device.

Further analysis of the data identified six key sub-themes based on what patients wanted from the device; control of the device, temperature of the device, cleanliness of the device, where the device should be located on the body, shape of the device and visual appearance of the device.

In addition, two key sub-themes were identified based on what HCPs would want from the device from a professional point of view: functionality of the device and universal compatibility of the device.

Finally, there were seven key sub-themes based on what HCPs believe patients would like; fitting the device into an object found in the treatment room, who controls the device, customisation of the device, where the device should be located on the body, non-clinical appearance of the device, temperature of the device and when to use the device. These themes are summarised in figure 1 and discussed in more detail below.

[Figure 1 near here]

For peer review only

PATIENT VIEWS

Control of the device:
Contrary to what HCPs thought patients might like, the patients reached strong consensus in favour of being able to control the device themselves. They highlighted that in times of uncertainty, the device would give them something to be in control of. Another popular opinion included a relative being able to control the device from home as many liked the idea of being able to interact with a loved one at a distance.

"That will make a big difference. And you've no control in these situations, so I think if you being able to control it probably would be quite reassuring."

Temperature of the device:

For peer review only - http://bmjopen.bmj.com/site/about/guidelines.xhtml

In a point of similarity to the HCPs, the most discussed feature of the device, and the most important feature for some, included the temperature of the device. The overall consensus was that the device should provide the patient with warmth.

"It's also temperature, when you hold someone's hand, it's a warm experience, whereas sometimes with some of the treatments you have, you feel quite cold and distant, and that's a bit of a strange one. It's like saying to someone it was all warm and fuzzy, it's a stupid thing to say, but actually temperature and touch and a feeling of wellbeing all go together, really."

Cleanliness of the device:

However, contrastingly to HCPs, only one patient expressed a desire for a clean device and stated that they would not want to use a device shaped like a glove if someone else had used it.

"…but then hygiene wise. If someone has used the glove, I wouldn't want to use it afterwards. But that's me."

Where the device should be located on the body:

Patient preferences for location on the body included a wide variety of locations: the hand, arm, shoulder, neck, head, leg, foot, buttocks, whole body and customisable – being able to use the same device in multiple locations. However, the most popular answer was using the device around the arm either because of a medical reason or personal preference.

"Maybe, for me, the arms, because I've got lymphedema, and that can be annoying."

Shape of the device:

Patients expressed interest in the shape of the device. Some suggested shaping the device, as a ball mimicking the shape of many conventional stress relieving devices, whist others suggested the shape of a teddy bear or comforting soft toy to help make associations with happier times.

"But for someone who is not so secure in that, something like that that you would just hold if you feel anxious. Because you don't know where they are. You know they've left you. But like the ball, it would just give you a degree of confidence as someone who isn't so secure in where they are."

Visual appearance of the device:

Finally, participants discussed making the device visually appealing to make the device look more interesting.

"Would it be possible to put lights or something inside it, so that it comes on… where the bits come out, colourful as well? "

Tactile sensation of the device:

For peer review only

Overall, the participants liked the idea of the device having a distinct tactile sensation, whether it is a pulsation, ripple, tapping, or vibration. The participants found these motions comforting and a form of distraction.

"It's clever isn't it? If you get it in the right pulse, it's just so calming. It is the ripple up and the ripple down, modulating."

HEALTHCARE PRACTITIONER VIEWS

Non-clinical appearance of the device:
All HCPs expressed a negative attitude towards the device appearing like a piece of clinical equipment. This was due to their belief that this would be off-putting to the patient. Two HCPS expressed that they felt blue was the correct colour for the device from a personal perspective.

"The clinical connotations, the association is always there for a blood pressure cuff."

Fitting the device into an object found in the treatment room:
The HCPs believed that patients would favour a device that was pre-fitted into an object found in the treatment room. They believed this would benefit patients who were nervous about disclosing their anxiety. From a professional point of view, the HCPs also believed that a piece of equipment designed to be left in-situ would be more efficient.

"You don't actually have to ask for something. You don't even have to say yes, I'd like something for my anxiety."

Control of the device:
The HCPs had different ideas about who the patient would most appreciate being in control of the device. Some HCPs believed that family members controlling the device would be the most comforting; enabling the patient to control the device could introduce a new anxiety on top of radiotherapy. Additionally, from a professional point of view, HCPs were concerned that if patients were given control of the device it could distract them from their workflow.

"Yes, definitely that element of someone else can control it is great. And I think, actually, a lot of patients would find that really comforting to know that their family, I don't know, their children, or something, was doing that I think."

Where the device should be located on the body:
One of the most widely held views among HCPs was that patients would want a flexible device that could be used in various locations on the body. If the device was not adaptable in terms of location, the second most favourable solution was to design a device for use on the feet. This was suggested both because of its similarity to a foot massage as well as because this is a location that was judged to be compatible with many radiotherapy set-ups.

For peer review only - http://bmjopen.bmj.com/site/about/guidelines.xhtml

"Maybe the ability for it to go... Adaptability, maybe, of different parts of the body or different equipment. Adaptability."

Temperature of the device:
Most HCPs believed that a warm device would be calming to a patient. However, a few HCPs disagreed and suggested that it would be good to adjust the temperature based on the needs of the patient.

"And, for me, I always like to make sure they're warm. Our treatment rooms can be quite cold. And if somebody is anxious, I don't know, I always think if I'm cold I'll be more anxious. So, it might be getting them an extra blanket, or making sure they've got an extra gown or something."

When to use the device:
Some HCPs believed patients would want to use the device in the waiting room to alleviate anxiety.

"And that's I think one of the first points where you can really tackle that anxiety as such and alleviate some of it."

Customisation of the device:
Overall, there was agreement on the fact that the device should be customisable in various ways in order to meet everyone's preferences. For example, patients should be able to choose whether they want to be able to feel the warmth, the tactile sensation employed and the position on the body the device is used.

DISCUSSION

Receiving cancer treatment can be a stress-provoking experience for patients for many reasons, including separation from their loved ones, an issue that is currently very pertinent (4). More specifically, with recent advances in radiotherapy, patients are required to spend an increasing amount of time in the treatment room, which may exacerbate the problem. Anxious patients may be managed pharmacologically but this is not appropriate for all patients and can be time consuming. On the non-pharmacological side, the number of interventions is limited (20). Further, the non-pharmacological interventions currently in place do not make up for the lack of physical interaction experienced by patients, instead focusing on distracting patients (for example through music).

We have identified key design aspects for an SAT device to alleviate the anxiety patients experience during radiotherapy and/or imaging. The implementation of such a device may help reduce costs through a smaller number of cancelled of procedures as well as less spent on medication to combat procedural anxiety.

For peer review only

Overall, we found that participant cohorts had mixed opinions on the design aspects of such a device. This could have been because of their different roles (HCP versus patient) and, therefore, different priorities – a phenomenon commented upon by Crowe et al (21).

Perhaps unsurprisingly, HCPs were concerned about the device not disrupting their workflow. For example, they emphasised designing an easily cleanable device, this is consistent with the literature which suggests interruptions may to their workload may contribute to medical errors (22). In contrast, patients and their carers often focused on anxiety-relieving features. This may be due to the high rates of anxiety experienced in these situations (4, 5). This can consequently lead to increased pain perception and an overall negative experience for the patient (23).

Control of the device was a controversial topic. Patients expressed the desire to have control of the device, whereas HCPs were keen for patients not to have control. HCP feared that patient control would lead to disruptions to the treatment session. Both groups agreed that allowing relatives some degree of control of the device would be good. This finding is supported by other studies that have shown the involvement of friends and family in radiotherapy treatment has a positive impact on patient care (24).

This controversy on control of the device warrants further exploration in future research. Specifically, how useful a device to reduce anxiety would be if it was controlled by the HCP against the patient's preference – perhaps relative control is a happy middle ground in this regard? As such, it is vital that future research incorporates further patient and public involvement and engagement, perhaps in the form of a project advisory group. Involving such an advisory group at all stages of the research could help to make sure the patient perspective is not neglected (25), ensuring any device is *both* amenable to patients as well as HCPs.

HCPs were divided regarding when the device should be used, with some suggesting patients might appreciate a calming device in the waiting room. Other HCPs suggested the treatment room, which tallies with patient views. The varying views are reflected in the literature which suggests that there is not a particular time that is anxiety-provoking for patients, and that anxiety can start a few days before the procedure and end days after (23, 26). Clarification on where exactly anxiety is most often experienced would be an ideal topic for future qualitative focus group studies.

If designed around the treatment room, HCPs suggest the device be left in-situ attached to the radiotherapy couch. The easy accessibility may benefit some patients who are anxious but do not report it. Patients from Black, Asian, and Minority Ethnic (BAME) communities are less likely to disclose and seek help for mental health issues but are at higher risk of mental health disorders. Reasons for not disclosing this information include either not recognising the symptoms or being in denial of mental health issues as a result of cultural beliefs (27, 28). The Department of Health has recognised the need to close the healthcare gap for BAME populations (26). Leaving the device in-situ would help address this need for those who are against disclosing such information.

For peer review only - http://bmjopen.bmj.com/site/about/guidelines.xhtml

Patients appreciated the simulated affective touch aspect of the device. Vibration and pulsation sensations were valued for their relaxing comfort and distraction, whereas 'stroking' motions were described as too human to be performed by a machine. The idea that simulated affective touch has the potential to relieve psychological distress is supported by the literature (13). However, it may be important not to make it too realistic as to fall into the hypothesised 'uncanny valley' (29) of imperfectly representing human features and actions.

Designing a device that is warm to touch was the idea most universally supported by both cohorts. Heat therapy has been shown to reduce the amount of serum cortisol and noradrenaline levels, hence downregulating the over-activated sympathetic nervous system in anxiety (30). Moreover, studies on various populations have demonstrated that heat therapy is an effective anxiolytic intervention in medical and non-medical situations (10, 11, 30).

HCPs emphasised the importance of a non-clinical visual appearance, particularly if applying the device to the arm. They were concerned patients may associate it with a blood pressure cuff, a device that can induce spike anxiety in certain patients (32). On the other hand, the only patient requirement was to make the device look visually appealing using lights and colour. Aesthetic factors have been suggested as important considerations in the healthcare setting, because they may influence whether patients will use the device (33).

HCPs often gave their views on device design from a professional standpoint, for example, by suggesting a flexible design on where it needs to be applied. This would allow patients to use the device wherever on the body they are having radiotherapy. Radiotherapy for head and neck cancer can be particularly anxiety-inducing due to the need for a close-fitting mask (12), and it may be prudent to make sure any device design accommodates these patients.

If this device proves to be beneficial for patients undergoing radiotherapy, it may have the potential to be used in other clinical settings to alleviate anxiety. For example, both cohorts had various suggestions about other occasions the device could be used including attending an MRI scan, chemotherapy and dementia care. MRI scans are known to cause anxiety (31); one study suggested that interventions including phone calls or videos to explain the situation significantly reduces anxiety before an MRI. However, studies have not explored interventions that could be used in-situ whilst having the MRI (12). Therefore, a priority may be finding the most appropriate device to be used in the medical setting.

The wider implications of this device are extensive. Overall, the literature suggests that there is a wider need for an appropriate anxiety alleviating device in the medical setting (10-12). In the case of a remote-controlled device operated by a relative, the project could have applicability to the COVID-19 pandemic where family members are unable to visit their loved ones in the hospital.

There were several limitations to this study. All participants were selected from a single geographical area which may have led to homogenous focus groups. Secondarily, if demographic data had been collected, it could have provided useful insight into the impact of ethnicity on design preferences for this device. The study also only considered adult

For peer review only

participants; future studies may consider how the design of an SAT device might differ for children.

The fact that this study uses two separate participant cohorts with different viewpoints (personal and professional) is a strength. Patient opinions are vital to any intervention & meeting patient needs will enhance satisfaction and therefore improve the overall clinical outcome (34). However, patient desires need to be balanced with HCP requirements as if the device was designed in a way that disrupted the treatment it would be unlikely to be implemented. Further qualitative research could be conducted on this topic (for example to resolve the issue of patient versus practitioner control of the device), with the use of a patient and public involvement project advisory group at all stages of the study to make sure the interests of both groups are equally represented (25, 35).

CONCLUSION

In conclusion, the results of this study support the idea that a tactile device has the potential to reduce procedural anxiety surrounding radiotherapy. Furthermore, the study identified which design features of an anxiety reducing tactile intervention would be valued by HCPs, patients and their carers. HCPs and patients agree on some features: a warm device which is flexible in where it can be used on the body (hence accommodating patients no matter where they are receiving radiotherapy). Areas where no consensus was reached, could be further explored and include greater patient and public involvement in the form of a project advisory group.

For peer review only

FIGURE/TABLE LEGENDS

Table 1. Description of focus groups and participants. A total of 30 participants were recruited, 13 radiotherapy patients, 10 healthcare practitioners and 3 carers. 4 other participants had not received radiotherapy or cared for someone who had experienced radiotherapy.

Figure 1- An outline of the overarching themes and the subsequent subthemes within. The Venn diagram demonstrates where the patients and HCPs agree and disagree with each other.

AUTHORS CONTRIBUTIONS

Hugo Hall and Yasmin Dhuga contributed equally to the composition of this paper and wish to be credited as co-lead authors if possible.

Alison McGregor and Helen McNair were the senior authors on this project and provided directorial oversight.

Caroline Yan Zheng and Elizabeth Joyce facilitated the focus groups alongside Helen McNair.

Caroline also provided feedback on draft manuscript versions alongside Gemma Clunie.

REFERENCES

1. International Agency for Research on Cancer. Cancer tomorrow - Estimated number of new cases from 2020 to 2040. https://gco.iarc.fr/tomorrow/en/dataviz/isotype (accessed 15 December 2020)
2. Delaney G, Jacob S, Featherstone C, Barton M. The role of radiotherapy in cancer treatment: estimating optimal utilization from a review of evidence-based clinical guidelines. *Cancer* 2005;104(6): 1129-37.
3. Begg AC, Stewart FA, Vens C. Strategies to improve radiotherapy with targeted drugs. *Nat Rev Cancer*. 2011;11(4):239-53.
4. Elsner K, Naehrig D, Halkett GKB, Dhillon HM. Reduced patient anxiety as a result of radiation therapist-led psychosocial support: a systematic review. *J Med Radiat Sci.* 2017;64(3):220-31.
5. Holmes N, Williamson K. A survey of cancer patients undergoing a radical course of radiotherapy, to establish levels of anxiety and depression. *J Radiother Pract.* 2008;7(2):89-98.
6. Royal College of Radiologists(2008) *On Target: Ensuring Geometric Accuracy in Radiotherapy*; p. 78. https://www.rcr.ac.uk/system/files/publication/field_publication_files/BFCO%2808%295_On_target.pdf (accessed 12 December 2020)
7. Chapman HA, Bernier D, Rusak B. MRI-related anxiety levels change within and between repeated scanning sessions. *Psychiatry Res.* 2010;182(2):160-4.

For peer review only

8. Murphy KJ, Brunberg JA. Adult claustrophobia, anxiety and sedation in MRI. *Magn Reson Imaging.* 1997;15(1):51-4.

9. National Health Service (2019) Diagnostic Imaging Dataset Annual Statistical Release 2018/2019. https://www.england.nhs.uk/statistics/wp-content/uploads/sites/2/2019/12/Annual-Statistical-Release-2018-19-PDF-1.9MB.pdf (accessed 21 December 2020)

10. Kim HJ, Kim JW, Park HS, Moon DG, Lee JG, Oh MM. The use of a heating pad to reduce anxiety, pain, and distress during cystoscopy in female patients. *Int Urogynecol J.* 2019;30(10):1705-10.

11. Hedén L, Von Essen L, Ljungman G. Randomized interventions for needle procedures in children with cancer. *Eur J Cancer Care (Engl).*2009;18(4):358-63.

12. Tugwell JR, Goulden N, Mullins P. Alleviating anxiety in patients prior to MRI: A pilot single-centre single-blinded randomised controlled trial to compare video demonstration or telephone conversation with a radiographer versus routine intervention. *Radiography (Lond).* 2018;24(2):122-9.

13. Triscoli C, Croy I, Steudte-Schmiedgen S, Olausson H, Sailer U. Heart rate variability is enhanced by long-lasting pleasant touch at CT-optimized velocity. *Biol Psychol*. 2017;128:71-81.

14. Parmar R, Brewer BB, Szalacha LA. Foot Massage, Touch, and Presence in Decreasing Anxiety during a Magnetic Resonance Imaging: A Feasibility Study. *J Altern Complement Med*. 2018;24(3):268-75.

15. Kolcaba K, Schirm V, Steiner R. Effects of hand massage on comfort of nursing home residents. *Geriatr Nurs*. 2006;27(2):85-91.

16. Von Mohr M, Kirsch LP, Fotopoulou A. The soothing function of touch: affective touch reduces feelings of social exclusion. *Sci Rep*. 2017;7(1):13516.

17. Faulkner SL, Trotter SP. Data Saturation. The International Encyclopedia of Communication Research Methods. 2017:1-2.

18. Braun V, Clarke V. Using thematic analysis in psychology. *Qual Res Psychol*. 2006;3(2):77-101.

19. Castleberry A, Nolen A. Thematic analysis of qualitative research data: Is it as easy as it sounds? *Curr Pharm Teach Learn*. 2018;10(6):807-15.

20. Forbes E, Baker AL, Britton B, Clover K, Skelton E, Oultram S, et al. Non-pharmacological approaches to procedural anxiety reduction for patients undergoing radiotherapy for cancer: systematic review protocol. *BMJ Open*. 2020;10(10):e035155.

21. Crowe S, Fenton M, Hall M, Cowan K, Chalmers I. Patients', clinicians' and the research communities' priorities for treatment research: there is an important mismatch. *Res Involv Engagem*. 2015;1(1):2.

22. Rivera-Rodriguez AJ, Karsh BT. Interruptions and distractions in healthcare: review and reappraisal. *Qual Saf Health Care*. 2010;19(4):304-12.

23. Appukuttan DP. Strategies to manage patients with dental anxiety and dental phobia: literature review. *Clin Cosmet Investig Dent*. 2016;8:35-50.

24. Mackenzie LJ, Sanson-Fisher RW, Carey ML, D'Este CA. Radiation oncology outpatient perceptions of patient-centred care: a cross-sectional survey. *BMJ Open*. 2013;3(2).

25. NIHR Involve. Briefing note three: Why involve members of the public in research? [Internet] https://www.invo.org.uk/posttyperesource/why-should-members-of-the-public-be-involved-in-research/ (accessed 24 January 2021)

For peer review only - http://bmjopen.bmj.com/site/about/guidelines.xhtml

26. Szczepura A. Access to health care for ethnic minority populations. *PMJ*. 2005;81(953):141.
27. Memon A, Taylor K, Mohebati LM, Sundin J, Cooper M, Scanlon T, et al. Perceived barriers to accessing mental health services among black and minority ethnic (BME) communities: a qualitative study in Southeast England. *BMJ Open*. 2016;6(11):e012337.
28. Weich S, Nazroo J, Sproston K, McManus S, Blanchard M, Erens B, et al. Common mental disorders and ethnicity in England: the EMPIRIC study. Psychol Med. 2004;34(8):1543-51.
29. Mathur MB, Reichling DB. Navigating a social world with robot partners: A quantitative cartography of the Uncanny Valley. *Cognition*. 2016;146:22-32.
30. Lee YH, Park BN, Kim SH. The effects of heat and massage application on autonomic nervous system. *Yonsei Med J.* 2011;52(6):982-9.
31. Stanley E, Cradock A, Bisset J, McEntee C, O'Connell MJ. Impact of sensory design interventions on image quality, patient anxiety and overall patient experience at MRI. *Br J Radiol.* 2016;89(1067):20160389.
32. Dillon KM, Seacat JD, Saucier CD, Doyle-Campbell CJ. Could Blood Pressure Phobia Go Beyond the White Coat Effect? *Am J Hypertens*. 2015;28(11):1306-9.
33. Lazard AJ, Watkins I, Mackert MS, Xie B, Stephens KK, Shalev H. Design simplicity influences patient portal use: the role of aesthetic evaluations for technology acceptance. *J Am Med Inform Assoc*. 2016;23(1):157-61.
34. Prakash B. Patient satisfaction. *J Cutan Aesthet Surg.* 2010;3(3):151-5.
35. Austin Z, Sutton J. Qualitative research: getting started. *Can J Hosp Pharm*. 2014;67(6):436-4

For peer review only

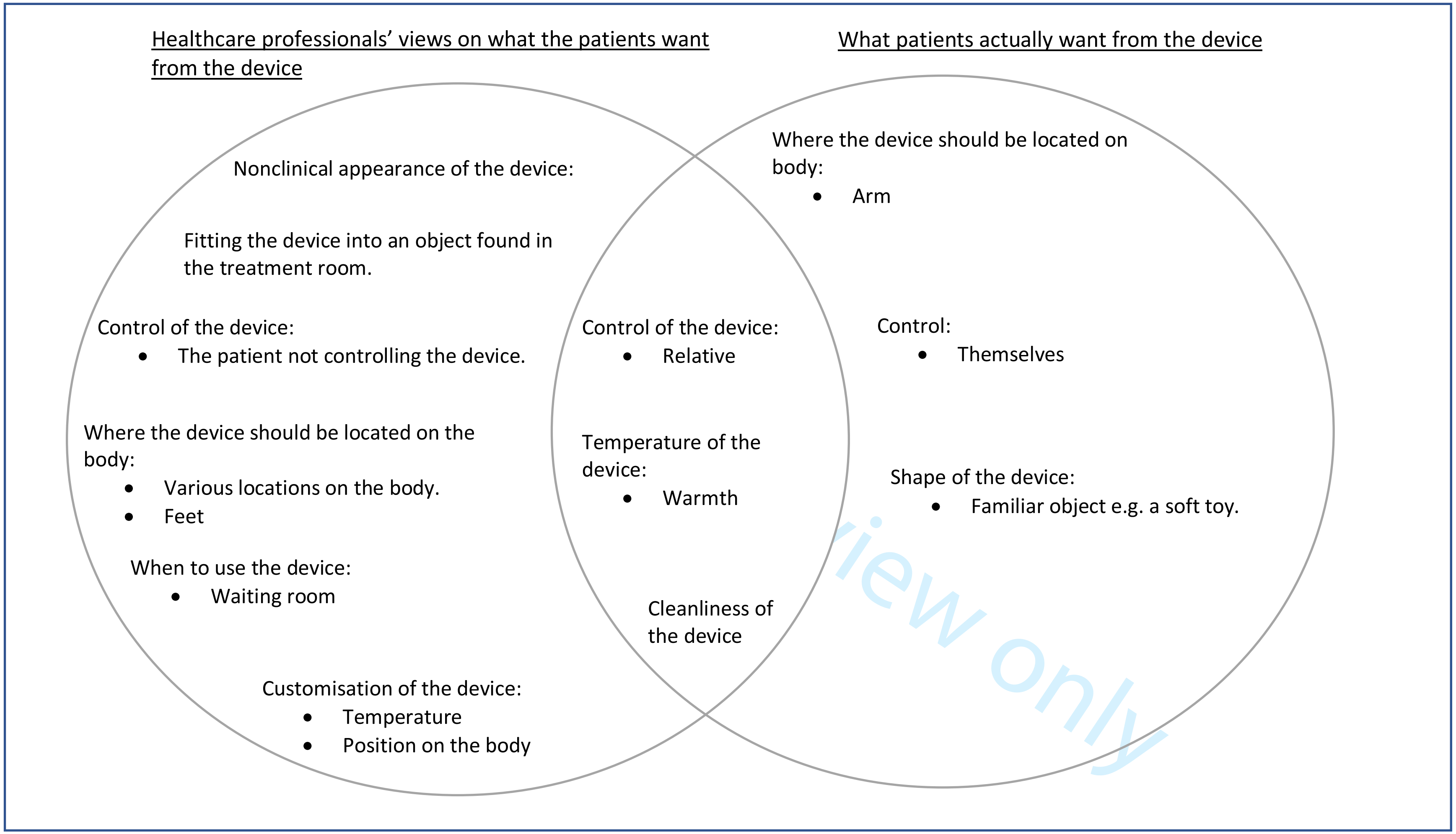

For peer review only - http://bmjopen.bmj.com/site/about/guidelines.xhtml

Standards for Reporting Qualitative Research (SRQR) as it applies to:

PATIENT AND PRACTITIONER PERSPECTIVES ON THE DESIGN OF A SIMULATED AFFECTIVE TOUCH DEVICE TO REDUCE PROCEDURAL ANXIETY ASSOCIATED WITH RADIOTHERAPY: A QUALITATIVE STUDY

O'Brien B.C., Harris, I.B., Beckman, T.J., Reed, D.A., & Cook, D.A. (2014). Standards for reporting qualitative research: a synthesis of recommendations. *Academic Medicine, 89(9)*, 1245-1251.

**No. Topic**	**Location in Article (page number)**
**Title and abstract**	
S1 Title	Page 1
S2 Abstract	Page 2
**Introduction**	
S3 Problem formulation	Page 4
S4 Purpose or research question	Page 4
**Methods**	
S5 Qualitative approach and research paradigm	Page 5
S6 Researcher characteristics and reflexivity	Pages 5-6
S7 Context	Page 5
S8 Sampling strategy	Page 5
S9 Ethical issues pertaining to human subjects	Page 5
S10 Data collection methods	Page 5
S11 Data collection instruments and technologies	Page 5
S12 Units of study	Page 5
S13 Data processing	Pages 5-6
S14 Data analysis	Page 6
S15 Techniques to enhance trustworthiness	Page 6
**Results/Findings**	
S16 Synthesis and interpretation	Pages 6-9
S17 Links to empirical data	Pages 6-9
**Discussion**	

For peer review only

S18 Integration with prior work, implications, transferability, and contribution(s) to the field	Pages 10-12
S19 Limitations	Page 12
**Other**	
S20 Conflicts of interest	Page 1
S21 Funding	Page 1

For peer review only

PATIENT AND PRACTITIONER PERSPECTIVES ON THE DESIGN OF A SIMULATED AFFECTIVE TOUCH DEVICE TO REDUCE PROCEDURAL ANXIETY ASSOCIATED WITH RADIOTHERAPY: A QUALITATIVE STUDY

Journal:	*BMJ Open*
Manuscript ID	bmjopen-2021-050288.R1
Article Type:	Original research
Date Submitted by the Author:	29-Sep-2021
Complete List of Authors:	Hall, Hugo ; King's College London; Imperial College London<br>Dhuga, Yasmin; Brighton and Sussex Medical School; Imperial College London<br>Yan Zheng, Caroline; Royal College of Art<br>Clunie, Gemma; Imperial College London Department of Surgery and Cancer, Sackler MSK Lab; Imperial College Healthcare NHS Trust, Speech and Language Therapy<br>Joyce, Elizabeth; Royal Marsden NHS Foundation Trust<br>McNair, Helen; Institute of Cancer Research, Radiotherapy and Imaging ; Royal Marsden NHS Foundation Trust<br>McGregor, Alison; Imperial College London, Dept of Surgery and Cancer
**Primary Subject Heading**:	Radiology and imaging
Secondary Subject Heading:	Oncology, Qualitative research
Keywords:	RADIOTHERAPY, QUALITATIVE RESEARCH, ONCOLOGY

I, the Submitting Author has the right to grant and does grant on behalf of all authors of the Work (as defined in the below author licence), an exclusive licence and/or a non-exclusive licence for contributions from authors who are: i) UK Crown employees; ii) where BMJ has agreed a CC-BY licence shall apply, and/or iii) in accordance with the terms applicable for US Federal Government officers or employees acting as part of their official duties; on a worldwide, perpetual, irrevocable, royalty-free basis to BMJ Publishing Group Ltd ("BMJ") its licensees and where the relevant Journal is co-owned by BMJ to the co-owners of the Journal, to publish the Work in this journal and any other BMJ products and to exploit all rights, as set out in our licence.

The Submitting Author accepts and understands that any supply made under these terms is made by BMJ to the Submitting Author unless you are acting as an employee on behalf of your employer or a postgraduate student of an affiliated institution which is paying any applicable article publishing charge ("APC") for Open Access articles. Where the Submitting Author wishes to make the Work available on an Open Access basis (and intends to pay the relevant APC), the terms of reuse of such Open Access shall be governed by a Creative Commons licence – details of these licences and which Creative Commons licence will apply to this Work are set out in our licence referred to above.

Other than as permitted in any relevant BMJ Author's Self Archiving Policies, I confirm this Work has not been accepted for publication elsewhere, is not being considered for publication elsewhere and does not duplicate material already published. I confirm all authors consent to publication of this Work and authorise the granting of this licence.

For peer review only

PATIENT AND PRACTITIONER PERSPECTIVES ON THE DESIGN OF A SIMULATED AFFECTIVE TOUCH DEVICE TO REDUCE PROCEDURAL ANXIETY ASSOCIATED WITH RADIOTHERAPY: A QUALITATIVE STUDY

Corresponding/senior authors:

Alison McGregor (a.mcgregor@imperial.ac.uk), Helen McNair (Helen.McNair@rmh.nhs.uk)

Co-authors:

Hugo Hall, Yasmin Dhuga, Caroline Yan Zheng, Gemma Clunie, Elizabeth Joyce, Helen McNair, Alison McGregor.

Hugo Hall and Yasmin Dhuga contributed equally to this paper.

Word count: 4650
Abstract – 226

Funding statement:

This research project was funded by the CRUK Convergence Science Centre at The Institute of Cancer Research, London, and Imperial College London (A26234)

We also acknowledge NHS funding to the NIHR Biomedical Research Centre at The Royal Marsden and The Institute of Cancer Research. The views expressed in this publication are those of the authors and not necessarily those of those of the NHS or the National Institute for Health Research.

Helen McNair is funded by a National Institute for Health Research and Health Education England (HEE/NIHR), Senior Clinical Lecturer award (ICA-SCL-2018-04-ST2-002).

Gemma Clunie is funded by the National Institute for Health Research (NIHR) Imperial Biomedical Research Centre and the NIHR Clinical Doctoral Research Fellowship Programme CDRF-2017-03-028/ Integrated Clinical Academic Programme.

Alison McGregor acknowledges support from the NIHR Imperial Biomedical Research Centre (BRC).

Disclosure Statement: No potential conflict of interest is reported by the authors.

Data Statement: Data (focus group transcripts) is held at Royal Marsden NHS Foundation Trust and can be accessed on request by contacting: Helen.McNair@rmh.nhs.uk

For peer review only

ABSTRACT

Objective The aim of this study was to elicit the views of relevant stakeholders on the design of a device using simulated affective touch to reduce procedural anxiety surrounding radiotherapy and imaging.

Design This qualitative study collected data from focus groups which were then analysed using inductive thematic analysis in line with Braun and Clarke's methods.

Participants and setting Twenty patients and carers were recruited, as well as ten healthcare practitioners involved in either delivering radiotherapy or imaging procedures.

Results Patients, carers and healthcare practitioners agreed on some aspects of the device design, such as ensuring the device is warm and flexible in where it can be used on the body. However, patient and healthcare practitioner cohorts had at times differing viewpoints. For example, healthcare practitioners provided professional perspectives and required easy cleaning of the device. Meanwhile patients focused on anxiety relieving factors, such as the tactile sensation of the device being either a vibration or pulsation. There was no consensus on who should control the device.

Conclusions The desired features of a simulated affective touch device have been investigated. Different priorities of patients, carers and healthcare practitioners were evident. Any design must incorporate such features as to appease both groups. Areas where no consensus was reached could be further explored, alongside including further patient and public involvement in the form of a project advisory group.

Key words: qualitative, focus groups, radiotherapy, procedural anxiety, tactile sensation

For peer review only

ARTICLE SUMMARY

STRENGTHS AND LIMITATIONS OF THIS STUDY

Strength This is the first qualitative study to explore whether a tactile intervention provides a useful tool in reducing procedural anxiety for radiotherapy patients.

Strength This study explores the opinions of two separate stakeholder cohorts, each with different viewpoints (personal and professional) around the design of the device.

Limitation All participants were selected from a single geographical area which may indicate a homogenous focus group composition.

Limitation Our study cannot be generalised to all individuals who receive radiotherapy, as this study only considered adult participants.

For peer review only

PATIENT AND PRACTITIONER PERSPECTIVES ON THE DESIGN OF A SIMULATED AFFECTIVE TOUCH DEVICE TO REDUCE PROCEDURAL ANXIETY ASSOCIATED WITH RADIOTHERAPY: A QUALITATIVE STUDY

INTRODUCTION

Currently 458,000 people live with cancer in the UK (1). Over 50% of these patients will be managed with radiotherapy (2, 3), which can cause procedural anxiety, with approximately 49% of patients experiencing anxiety and psychological distress (4, 5). Procedural anxiety refers to excessive worry or fear of medical procedures (6) and is a phenomenon exacerbated with new developments in radiotherapy, for example stereotactic and adaptive radiotherapy, that require long treatment times.

High levels of distress during radiotherapy can directly impact the accuracy and efficacy of the procedure (7). However, the consequences of procedural anxiety extend beyond radiotherapy and include diagnostic imaging, and other invasive procedures performed on the conscious patient. Between 2-5% of magnetic resonance imaging (MRI) scans are terminated due to procedural anxiety (8, 9); this equates to a significant financial cost for the NHS considering that 3.4 million MRI scans were conducted between 2017 to 2018 (10).

Pre-existing interventions to manage procedural anxiety are limited and there is a need for an appropriate anxiety alleviating strategy or device in medical settings (11-13). Techniques such as verbal communication or music may distract the patient but do not address the reported sense of isolation associated with separation from caregivers or families. Sedation is commonly used to tackle procedural anxiety in the context of MRI but has implications for cost, risk and time (9). This has implications in the context of daily radiotherapy treatment. On top of increased risk to the patient, sedation requires the presence of an anaesthetics team which has time implications for the treatment, facility and hospital resources.

Research indicates that human affective (or empathetic) touch 'provides both psychological and physical comfort' Peled-Avron et al (14). However, even prior to restrictions imposed on visiting by the COVID-19 pandemic, relatives and carers have been prohibited from being in the radiotherapy room. As such, this research focuses on patient's perspectives of a simulated affective touch (SAT) device - a haptic device that uses tactile simulations to mimic attributes of human affective touch.

Such tactile interventions have not been widely explored as a tool to reduce procedural anxiety in radiotherapy patients. However, haptic devices that simulate attributes of human affective touch, such as gentle stroking, have been shown to produce pleasant sensation (15), relieve psychological distress (16) and reduce the sense of social isolation associated with being apart from caregivers (17-19). Thus, it is postulated that SAT can provide a non-invasive intervention to combat procedural anxiety surrounding radiotherapy due to their ability to reproduce natural touch (20).

For peer review only - http://bmjopen.bmj.com/site/about/guidelines.xhtml

The following paper aims to investigate both patient and healthcare professional (HCP) views on the creation of a calming device using SAT to combat induced procedural anxiety. Patients were asked about what aspects of any SAT device they would find calming. Carers play a vital role in supporting their loved ones through difficult times, a view endorsed by a recent study MacMillan survey (21) which reported 74% of carers supported their loved ones by talking and listening to them. Therefore, we can assume they have insight on the impact of medical procedures on their loved ones and also have a role in the requirements of such a device. Finally, the views of HCPs as key stakeholders in any potential device were collected to ensure future clinical usability and translation. HCPs were able to advise both on professional requirements (e.g. cleanability), as well as what they thought patients might appreciate about any calming SAT device. These insights will facilitate the future development of a design brief for an SAT prototype device to reduce procedural anxiety which is suitable for clinical testing.

METHODS

Study Design

This was a qualitative co-design study using focus groups to elicit the views of two distinct categories of participants, patients and healthcare professionals, on the creation of an SAT device for use during radiotherapy interventions.

The purpose of the focus group was to establish participant thoughts around the experience of soft robotic tactile intervention probing artefacts. First, we established the ground rules and agenda of the focus group. Four probing artefacts in different shapes, touch patterns and interaction options were then introduced to the group to serve as prompts. This enabled participants to engage in discussion around the desirable and undesirable features of a tactile intervention device.

Example questions included:

A) How participants feel about the sensations
B) Whether they would consider the device useful in helping to alleviate possible anxiety during the care journey
C) Where during the care journey they think this device would be useful
D) What sort of sensations they liked and would prefer

We adopted a phenomenological approach using inductive thematic analysis. This allowed us to focus on how best to use the product design to counter the phenomena of procedural anxiety around radiotherapy and imaging.

The study protocol was approved by Greater Manchester South Research Ethics Committee, and permission to conduct the research was granted by Health and Care Research Wales. REC reference: 19/NW/0607, Protocol number: CCR5133, IRAS project ID: 266016.

For peer review only

Participants and Setting

Participants were recruited from two groups of stakeholders: one comprised both patients and carers, the other group comprised health care professionals. Patients and carers were invited to participate as experts on the lived experience of having radiotherapy and/or imaging procedures; this allowed us to explore circumstances and situations that may have created anxiety and to provide ideas on how to manage these. The views of healthcare practitioners (HCPs) involved in delivering radiotherapy or imaging procedures were important as key stakeholders if the device is to become part of clinical practice.

Convenience sampling was used to select both participant groups. Inclusion criteria stipulated participants were aged over 18 years, while those patients who were considered too unwell (by self-report or in consultation with clinical staff) were excluded. Those who were unable to understand English without an interpreter were also excluded. Patients and their carers were contacted using flyers placed in radiotherapy treatment and pre-treatment waiting rooms, while HCPs were internally recruited by departmental email. Patients from the local Biomedical Research Centre (BRC) Patient and Public Involvement (PPI) groups were also emailed. Information sheets were distributed to interested patients and carers who then gave written informed consent to join the study. Informed written consent was also obtained from HCPs participating in the study.

Data Collection

A total of six focus groups took place at the Royal Marsden Hospital. The total number of participants was 30 (table 1), with four patient and caregiver groups (two groups with four participants, two groups with six participants) and two HCP groups (five participants each). Of the 10 HCPs, nine were therapeutic radiographers (eight involved in the planning or treatment of radiotherapy patients and one educational lead) and one was a diagnostic radiographer working mainly in magnetic resonance imaging. The patient and caregiver focus groups lasted on average 83 minutes (ranging from 82 – 85 minutes). The HCP focus groups lasted on average 49 minutes (ranging from 48 – 50 minutes). Recruitment was stopped after six focus groups when initial analysis of the themes demonstrated no new emerging themes, indicative of saturation (22).

For peer review only

	Patient had experience of radiotherapy	Cared for someone with radiotherapy experience	Other*	Worked as a healthcare practitioner in radiotherapy
**Totals**	13	3	4	10
**Gender**				
**Male**	4	1	3	-
**Female**	9	2	1	10
**Age**				
**18-25**	-	-		1
**26-35**	-	-		5
**36-45**	-	-		2
**46-55**	2	-		2
**56-65**	7	1	2	-
**66-75**	3	2	2	-
**76-85**	1	-		-

Focus groups were recorded and transcribed verbatim for analysis. Three qualitative researchers of mixed experience and a design researcher were responsible for facilitating the focus groups. All three qualitative researchers had no prior relationship with the patient participants but two of them were well known to the HCP cohort. To address this potential limitation, an independent researcher led all the focus groups - the researchers known to the participants only attended one of the groups. The varied comments and depth of discussion implies that that there was sufficient freedom for articulating thoughts in each of the groups. In addition, the design researcher was not known to any participants.

Data Analysis

Thematic analysis was used to identify themes and sub-themes inductively within the focus group data. In line with Braun and Clarke's (23) methods, the initial step was for researchers to familiarise themselves with the interview recordings. Next, transcripts were analysed using QSR NVivo Release 1.1 and all information relating to calming measures was coded. Each transcript was double coded (once by the design researcher and once by the medical researchers) allowing for multiple perspectives on the analysis. An independent researcher then reviewed the codes to increase inter-coder reliability and the trustworthiness of the

For peer review only

results (24). Periodic meetings about the analysis with the whole project team led to iterations and finally the collation of the codes into themes and sub-themes.

Patient and Public Involvement

The concept was presented at initial planning stages to two Royal Marsden NHS PPI groups. Positive feedback was received which enabled us to proceed with study.

RESULTS

Three overarching themes emerged from analysis of the data: patient views on what they want from the device, HCP views on the device and HCP views on what they believe patients want from the device.

Further analysis of the data identified six key sub-themes based on what patients wanted from the device; control of the device, temperature of the device, cleanliness of the device, where the device should be located on the body, shape of the device and visual appearance of the device.

In addition, two key sub-themes were identified based on what HCPs would want from the device from a professional point of view: functionality of the device and universal compatibility of the device.

Finally, there were seven key sub-themes based on what HCPs believe patients would like; fitting the device into an object found in the treatment room, who controls the device, customisation of the device, where the device should be located on the body, non-clinical appearance of the device, temperature of the device and when to use the device. These themes are summarised in figure 1 and discussed in more detail below.

[Figure 1 near here]

PATIENT VIEWS

Control of the device:
Contrary to what HCPs thought patients might like, the patients reached strong consensus in favour of being able to control the device themselves. They highlighted that in times of uncertainty, the device would give them something to be in control of. Another popular opinion included a relative being able to control the device from home as many liked the idea of being able to interact with a loved one at a distance.

For peer review only - http://bmjopen.bmj.com/site/about/guidelines.xhtml

"That will make a big difference. And you've no control in these situations, so I think if you being able to control it probably would be quite reassuring."
-P7: Cared for someone with radiotherapy experience

Temperature of the device:
In a point of similarity to the HCPs, the most discussed feature of the device, and the most important feature for some, included the temperature of the device. The consensus was that the device should provide the patient with warmth.

"It's also temperature, when you hold someone's hand, it's a warm experience, whereas sometimes with some of the treatments you have, you feel quite cold and distant, and that's a bit of a strange one. It's like saying to someone it was all warm and fuzzy, it's a stupid thing to say, but actually temperature and touch and a feeling of wellbeing all go together, really."
-P17: Patient had experience of radiotherapy

Cleanliness of the device:
Contrastingly to HCPs, only one patient expressed a desire for a clean device and stated that they would not want to use a device shaped like a glove if someone else had used it.

"…but then hygiene wise. If someone has used the glove, I wouldn't want to use it afterwards. But that's me."
-P7: Cared for someone with radiotherapy experience

Where the device should be located on the body:
Patient preferences for location on the body included a wide variety of locations: the hand, arm, shoulder, neck, head, leg, foot, buttocks, whole body and customisable – being able to use the same device in multiple locations. However, the most popular answer was using the device around the arm either because of a medical reason or personal preference.

"Maybe, for me, the arms, because I've got lymphedema, and that can be annoying."
-P10: Other (imaging but not radiotherapy experience)

Shape of the device:
Patients expressed interest in the shape of the device. The ball shaped probing artifact received good feedback with several participants expressing positive sentiments towards it based on its shape. Other participants suggested the shape of a teddy bear or comforting soft toy to help make associations with happier times.

"the ball, it would just give you a degree of confidence as someone who isn't so secure in where they are."
-P19: Patient had experience of radiotherapy

Visual appearance of the device:
Finally, participants discussed making the device visually appealing to make the device look more interesting.

For peer review only - http://bmjopen.bmj.com/site/about/guidelines.xhtml

"Would it be possible to put lights or something inside it, so that it comes on… where the bits come out, colourful as well? "
-P12: Patient had experience of radiotherapy

Tactile sensation of the device:
Overall, the participants liked the idea of the device having a distinct tactile sensation, whether it is a pulsation, ripple, tapping, or vibration. The participants found these motions comforting and a form of distraction.

"It's clever isn't it? If you get it in the right pulse, it's just so calming. It is the ripple up and the ripple down, modulating."
-P3: Patient had experience of radiotherapy

HEALTHCARE PRACTITIONER VIEWS

Non-clinical appearance of the device:
All HCPs expressed a negative attitude towards the device appearing like a piece of clinical equipment. This was due to their belief that this would be off-putting to the patient. Two HCPS expressed that they felt blue was the correct colour for the device from a personal perspective.

"you wouldn't want it to look too clinical"
-P27: radiographer

"because of the patient age group. They might think, oh, you're taking my blood pressure."
-P29: radiographer

Fitting the device into an object found in the treatment room:
The HCPs believed that patients would favour a device that was pre-fitted into an object found in the treatment room. They believed this would benefit patients who were nervous about disclosing their anxiety. From a professional point of view, the HCPs also believed that a piece of equipment designed to be left in-situ would be more efficient.

"You don't actually have to ask for something. You don't even have to say yes, I'd like something for my anxiety."
-P23: radiographer

Control of the device:
The HCPs had different ideas about who the patient would most appreciate being in control of the device. Some HCPs believed that family members controlling the device would be the most comforting; enabling the patient to control the device could introduce a new anxiety on top of radiotherapy. Additionally, from a professional point of view, HCPs were concerned that if patients were given control of the device, it could distract the HCP from their workflow due to a lack of co-operation from the patient when required.

For peer review only

"Yes, definitely that element of someone else can control it is great. And I think, actually, a lot of patients would find that really comforting to know that their family, I don't know, their children, or something, was doing that I think."
-P26: radiographer

"I just wonder if [controlling the device] introduces too many elements... They're overwhelmed already...He's relaxed, then he switches it off, then in treatment... They then panic because they want it back on. I think it introduces too many overwhelming factors."
-P28: radiographer

Where the device should be located on the body:
One of the most widely held views among HCPs was that patients would want a flexible device that could be used in various locations on the body. If the device was not adaptable in terms of location, the second most favourable solution was to design a device for use on the feet. This was suggested both because of its similarity to a foot massage as well as because this is a location that was judged to be compatible with many radiotherapy set-ups.

"Maybe the ability for it to go... Adaptability, maybe, of different parts of the body or different equipment. Adaptability."
-P28: radiographer

Cleanliness of the device:
HCP were keen to have a device that was easily cleanable and wipeable, with one group listing it as their top feature required when considering the practicality of an SAT device for use in radiotherapy. This research was conducted pre-COVID-19 pandemic, and so this feature would likely be of even greater importance now that infection control procedures and the disinfection of all equipment post-use have been brought into such sharp focus.

"Everything would need to be wipeable."
-P27: radiographer

"it has to pass infection control"
-P25: radiographer

"Facilitator: Practicalities. Where would you fit the practicalities?
P27: I would say that it's cleanable being up at the top.
P28: It has to be top, yes..."
-P27, P28: both radiographers

Temperature of the device:
Most HCPs believed that a warm device would be calming to a patient. However, a few HCPs disagreed and suggested that it would be good to adjust the temperature based on the needs of the patient.

"And, for me, I always like to make sure they're warm. Our treatment rooms can be quite cold. And if somebody is anxious, I don't know, I always think if I'm cold I'll be more anxious.

For peer review only - http://bmjopen.bmj.com/site/about/guidelines.xhtml

So, it might be getting them an extra blanket, or making sure they've got an extra gown or something."
-P25: radiographer

When to use the device:
Some HCPs believed patients would want to use the device in the waiting room to alleviate anxiety.

"And that's I think one of the first points where you can really tackle that anxiety as such and alleviate some of it."
-P28: radiographer

Customisation of the device:
Overall, there was agreement on the fact that the device should be customisable in various ways in order to meet everyone's preferences. For example, patients should be able to choose whether they want to be able to feel the warmth, the tactile sensation employed and the position on the body the device is used.

DISCUSSION

Receiving cancer treatment can be a stress-provoking experience for patients for many reasons, including separation from their loved ones, an issue that is currently very pertinent (4). With recent advances in radiotherapy, patients are required to spend an increasing amount of time in the treatment room, which may exacerbate such problems. Anxious patients may be managed pharmacologically but this is not appropriate for all patients and can be time consuming. On the non-pharmacological side, the number of interventions is limited (25). Further, the non-pharmacological interventions currently in place do not make up for the lack of physical interaction experienced by patients, instead focusing on distracting patients (for example through music).

We have identified key design aspects for an SAT device to alleviate the anxiety patients experience during radiotherapy and/or imaging. The implementation of such a device may help reduce costs through a smaller number of cancelled procedures as well as less spent on medication to combat procedural anxiety.

Overall, we found that participant cohorts had mixed opinions on the design aspects of such a device. This could have been because of their different roles (HCP versus patient) and, therefore, different priorities – a phenomenon commented upon by Crowe et al (26).

Perhaps unsurprisingly, HCPs were concerned about the device not disrupting their workflow. For example, they emphasised designing an easily cleanable device, this is consistent with the literature which suggests interruptions to their workload may contribute to medical errors (27). In contrast, patients and their carers often focused on anxiety-

For peer review only

relieving features. This may be due to the high rates of anxiety experienced during radiotherapy (4, 5).

Control of the device was a controversial topic. Patients expressed the desire to have control of the device, whereas HCPs were keen for patients not to have control. HCP feared that patient control would lead to disruptions to the treatment session. Both groups agreed that allowing relatives some degree of control of the device would be good. This finding is supported by other studies that have shown the involvement of friends and family in radiotherapy treatment has a positive impact on patient care (28).

This controversy on control of the device warrants further exploration in future research. Specifically, how useful a device to reduce anxiety would be if it was controlled by the HCP against the patient's preference – relative control may provide a happy middle ground in this regard. It is vital that future research incorporates further patient and public involvement and engagement, perhaps in the form of a project advisory group. Involving such an advisory group at all stages of the research could help to make sure the patient perspective is not neglected (29), ensuring any device is both amenable to patients as well as HCPs.

HCPs were divided regarding when the device should be used, with some suggesting patients might appreciate a calming device in the waiting room. Other HCPs suggested the treatment room, which conforms with patient views. The varying views are reflected in the literature which suggests that there is not a particular time that is anxiety-provoking for patients, and that anxiety can start a few days before the procedure and end days after (30, 31). Clarification on where exactly anxiety is most often experienced would be an ideal topic for future qualitative focus group studies.

If designed around the treatment room, HCPs suggest the device be left in-situ attached to the radiotherapy couch. The easy accessibility may benefit some patients who are anxious but do not report it. Patients from Black, Asian, and Minority Ethnic (BAME) communities are less likely to disclose and seek help for mental health issues but are at higher risk of mental health disorders. Reasons for not disclosing this information include either not recognising the symptoms or being in denial of mental health issues as a result of cultural beliefs (32, 33). The Department of Health has recognised the need to close the healthcare gap for BAME populations (31). Leaving the device in-situ would help address procedural anxiety for those who are against disclosing that they are anxious.

Patients appreciated the simulated affective touch aspect of the device. Vibration and pulsation sensations were valued for their relaxing comfort and distraction, whereas 'stroking' motions were described as too human to be performed by a machine. The idea that simulated affective touch has the potential to relieve psychological distress is supported by the literature (16). However, it may be important not to make it too realistic as to fall into the hypothesised 'uncanny valley' (34) of imperfectly representing human features and actions.

Designing a device that is warm to touch was the idea most universally supported by both participant cohorts. Heat therapy has been shown to reduce the amount of serum cortisol

For peer review only

and noradrenaline levels, hence downregulating the over-activated sympathetic nervous system in anxiety (35). Moreover, studies on various populations have demonstrated that heat therapy is an effective anxiolytic intervention in medical and non-medical situations (11, 12, 35).

HCPs emphasised the importance of a non-clinical visual appearance, particularly if applying the device to the arm. They were concerned patients may associate it with a blood pressure cuff, a device that can induce spike anxiety in certain patients (36). On the other hand, the only patient requirement was to make the device look visually appealing using lights and colour. Aesthetic factors have been suggested as important considerations in the healthcare setting because they may influence whether patients will use the device (37).

HCPs often gave their views on device design from a professional standpoint, for example, by suggesting a design which can be applied to multiple different areas of the body. This would allow patients to use the device wherever on the body they are having radiotherapy. Radiotherapy for head and neck cancer can be particularly anxiety-inducing due to the need for a close-fitting mask (13), and it may be prudent to make sure any device design accommodates these patients.

If this device proves to be beneficial for patients undergoing radiotherapy, it may have the potential to be used in other clinical settings to alleviate anxiety. For example, both cohorts had various suggestions about other occasions the device could be used including attending an MRI scan, chemotherapy and dementia care. MRI scans are known to cause anxiety (38), and there have been investigations into interventions prior to the procedure to alleviate anxiety. Interventions have included calls to or videos for patients to watch which explain the procedure (13). Whilst reducing anxiety levels, these interventions cannot be delivered in-situ during the procedure (13), which is something that an SAT device could achieve.

More generally, the literature suggests that there is a need for an appropriate anxiety alleviating device in the medical setting (11-13) but a solution has not previously been found. The case for a remote-controlled SAT device operated by a relative, has applicability to the COVID-19 pandemic where imposed social distancing (maintaining a 2-meter distance from others) and many hospitals prohibiting in-person visitors lead to high levels of distress amongst patients and their families.

There are several limitations to this study. All participants were selected from a single geographical area which may have led to homogenous focus groups. Secondly, if demographic data had been collected, it could have provided useful insight into the impact of ethnicity on design preferences for this device. The study also only considered adult participants; future studies may consider how the design of an SAT device might differ for children.

The fact that this study uses two separate participant cohorts with different viewpoints (personal and professional) is a strength. Patient opinions are vital to any intervention & meeting patient needs will enhance satisfaction and therefore improve the overall clinical outcome (39). However, patient desires need to be balanced with HCP requirements as if the device was designed in a way that disrupted the treatment it would be unlikely to be

For peer review only

implemented. Further qualitative research could be conducted on this topic with the use of a patient and public involvement project advisory group at all stages of the study to make sure the interests of both groups are equally represented (29, 40).

CONCLUSION

In conclusion, we have identified there is a lack of data around the benefit of using tactile interventions as a tool to reduce procedural anxiety in radiotherapy patients. This study identified which design features of an anxiety reducing tactile intervention would be valued by HCPs, patients and their carers. HCPs and patients agree on some features: a warm device which is flexible in where it can be used on the body (hence accommodating patients no matter where they are receiving radiotherapy). Both agreed that relatives should have some control over the device. HCPs wanted to ensure the device can easily be cleaned while patients were keen for the vibration and pulsation sensations to be used to put them at ease. Areas where no consensus was reached could be further explored as well as including greater patient and public involvement in the form of a project advisory group.

The authors intend to incorporate the desirable design features identified from this study to develop a design brief for a soft robotic device to alleviate procedural anxiety. The next stage would be to create a prototype device to test the usability and utility of this concept.

For peer review only

FIGURE/TABLE LEGENDS

Table 1. Description of focus groups and participants. A total of 30 participants were recruited, 13 radiotherapy patients, 10 healthcare practitioners and three carers. Four other participants had not received radiotherapy or cared for someone who had experienced radiotherapy. 'Other' refers to cancer patients recruited from the BRC Patient and public involvement group *who had received imaging (e.g. MRI) but not radiotherapy.*

Figure 1- An outline of the overarching themes and the subsequent subthemes within. The Venn diagram demonstrates where the patients and HCPs agree and disagree with each other.

AUTHORS CONTRIBUTIONS

Hugo Hall and Yasmin Dhuga contributed equally to the composition of this paper as well as the thematic analysis of the data and wish to be credited as co-lead authors if possible.

Alison McGregor and Helen McNair were the senior authors on this project and provided directorial oversight.

Helen McNair, Alison McGregor and Caroline Yan Zheng developed the study design and focus group guide.

Caroline Yan Zheng and Elizabeth Joyce and Gemma Clunie facilitated the focus groups alongside Helen McNair.

Caroline also conducted initial data analysis and, alongside Gemma Clunie, provided feedback on draft manuscript versions.

REFERENCES

1. International Agency for Research on Cancer. Cancer tomorrow - Estimated number of new cases from 2020 to 2040. https://gco.iarc.fr/tomorrow/en/dataviz/isotype [accessed 15th December 2020].
2. Delaney G, Jacob S, Featherstone C, Barton M. The role of radiotherapy in cancer treatment: estimating optimal utilization from a review of evidence-based clinical guidelines. *Cancer* 2005;104(6): 1129-37.
3. Begg AC, Stewart FA, Vens C. Strategies to improve radiotherapy with targeted drugs. *Nat Rev Cancer*. 2011;11(4):239-53.
4. Elsner K, Naehrig D, Halkett GKB, Dhillon HM. Reduced patient anxiety as a result of radiation therapist-led psychosocial support: a systematic review. *J Med Radiat Sci.* 2017;64(3):220-31.

For peer review only

5. Holmes N, Williamson K. A survey of cancer patients undergoing a radical course of radiotherapy, to establish levels of anxiety and depression. *J Radiother Pract.* 2008;7(2):89-98.
6. Choy Y. Acute procedure anxiety in adults: course, screening, assessment, and differential diagnosis: UpToDate, 2019. Available: https://www.uptodate.com/contents/acute-procedure-anxiety-in-adults-course-screening-assessment-and-differential-diagnosis [Accessed 18 Mar 2019].
7. Royal College of Radiologists (2008) *On Target: Ensuring Geometric Accuracy in Radiotherapy*; p. 78. https://www.rcr.ac.uk/system/files/publication/field_publication_files/BFCO%2808%295_On_target.pdf [accessed 12 December 2020].
8. Chapman HA, Bernier D, Rusak B. MRI-related anxiety levels change within and between repeated scanning sessions. *Psychiatry Res.* 2010;182(2):160-4.
9. Murphy KJ, Brunberg JA. Adult claustrophobia, anxiety and sedation in MRI. *Magn Reson Imaging.* 1997;15(1):51-4.
10. National Health Service (2019) Diagnostic Imaging Dataset Annual Statistical Release 2018/2019. https://www.england.nhs.uk/statistics/wp-content/uploads/sites/2/2019/12/Annual-Statistical-Release-2018-19-PDF-1.9MB.pdf [accessed 21 December 2020].
11. Kim HJ, Kim JW, Park HS, Moon DG, Lee JG, Oh MM. The use of a heating pad to reduce anxiety, pain, and distress during cystoscopy in female patients. *Int Urogynecol J.* 2019;30(10):1705-10.
12. Hedén L, Von Essen L, Ljungman G. Randomized interventions for needle procedures in children with cancer. *Eur J Cancer Care (Engl).*2009;18(4):358-63.
13. Tugwell JR, Goulden N, Mullins P. Alleviating anxiety in patients prior to MRI: A pilot single-centre single-blinded randomised controlled trial to compare video demonstration or telephone conversation with a radiographer versus routine intervention. *Radiography (Lond).* 2018;24(2):122-9.
14. Peled-Avron L, Levy-Gigi E, Richter-Levin G, Korem N, Shamay-Tsoory SG. The role of empathy in the neural responses to observed human social touch. *Cognit Affect Behav Neurosci*. 2016;16(5):802–813.
15. Zheng, C. Y. et al. (2021) 'Comparing soft robotic affective touch to human and brush affective touch', in *2021 IEEE World Haptics Conference (WHC)*. [Online]. July 2021 pp. 352–352.
16. Triscoli C, Croy I, Steudte-Schmiedgen S, Olausson H, Sailer U. Heart rate variability is enhanced by long-lasting pleasant touch at CT-optimized velocity. *Biol Psychol*. 2017;128:71-81.
17. Parmar R, Brewer BB, Szalacha LA. Foot Massage, Touch, and Presence in Decreasing Anxiety during a Magnetic Resonance Imaging: A Feasibility Study. *J Altern Complement Med*. 2018;24(3):268-75.
18. Kolcaba K, Schirm V, Steiner R. Effects of hand massage on comfort of nursing home residents. *Geriatr Nurs*. 2006;27(2):85-91.
19. Von Mohr M, Kirsch LP, Fotopoulou A. The soothing function of touch: affective touch reduces feelings of social exclusion. *Sci Rep*. 2017;7(1):13516.
20. Goldsworthy, S. et al. The potential for haptic touch technology to supplement human empathetic touch during radiotherapy. Journal of Medical Imaging and Radiation Sciences. [Online]. 2020; 51 (4, Supplement); S39–S43.

For peer review only - http://bmjopen.bmj.com/site/about/guidelines.xhtml

21. Macmillan Cancer Support. Understanding the numbers, needs and experiences of cancer carers in the UK. https://www.macmillan.org.uk/_images/carers-of-people-with-cancer_tcm9-282780.pdf [accessed 9th September 2021].
22. Faulkner SL, Trotter SP. Data Saturation. The International Encyclopedia of Communication Research Methods. 2017:1-2.
23. Braun V, Clarke V. Using thematic analysis in psychology. *Qual Res Psychol*. 2006;3(2):77-101.
24. Castleberry A, Nolen A. Thematic analysis of qualitative research data: Is it as easy as it sounds? *Curr Pharm Teach Learn*. 2018;10(6):807-815.
25. Forbes E, Baker AL, Britton B, Clover K, Skelton E, Oultram S, et al. Non-pharmacological approaches to procedural anxiety reduction for patients undergoing radiotherapy for cancer: systematic review protocol. *BMJ Open*. 2020;10(10):e035155.
26. Crowe S, Fenton M, Hall M, Cowan K, Chalmers I. Patients', clinicians' and the research communities' priorities for treatment research: there is an important mismatch. *Res Involv Engagem*. 2015;1(1):2.
27. Rivera-Rodriguez AJ, Karsh BT. Interruptions and distractions in healthcare: review and reappraisal. *Qual Saf Health Care*. 2010;19(4):304-12.
28. Mackenzie LJ, Sanson-Fisher RW, Carey ML, D'Este CA. Radiation oncology outpatient perceptions of patient-centred care: a cross-sectional survey. *BMJ Open*. 2013;3(2).
29. NIHR Involve. Briefing note three: Why involve members of the public in research? [Internet] https://www.invo.org.uk/posttyperesource/why-should-members-of-the-public-be-involved-in-research/ [accessed 24 January 2021].
30. Appukuttan DP. Strategies to manage patients with dental anxiety and dental phobia: literature review. *Clin Cosmet Investig Dent*. 2016;8:35-50.
31. Szczepura A. Access to health care for ethnic minority populations. *PMJ*. 2005;81(953):141.
32. Memon A, Taylor K, Mohebati LM, Sundin J, Cooper M, Scanlon T, et al. Perceived barriers to accessing mental health services among black and minority ethnic (BME) communities: a qualitative study in Southeast England. *BMJ Open*. 2016;6(11).
33. Weich S, Nazroo J, Sproston K, McManus S, Blanchard M, Erens B, et al. Common mental disorders and ethnicity in England: the EMPIRIC study. Psychol Med. 2004;34(8):1543-1551.
34. Mathur MB, Reichling DB. Navigating a social world with robot partners: A quantitative cartography of the Uncanny Valley. *Cognition*. 2016;146:22-32.
35. Lee YH, Park BN, Kim SH. The effects of heat and massage application on autonomic nervous system. *Yonsei Med J.* 2011;52(6):982-989.
36. Dillon KM, Seacat JD, Saucier CD, Doyle-Campbell CJ. Could Blood Pressure Phobia Go Beyond the White Coat Effect? *Am J Hypertens*. 2015;28(11):1306-1309.
37. Lazard AJ, Watkins I, Mackert MS, Xie B, Stephens KK, Shalev H. Design simplicity influences patient portal use: the role of aesthetic evaluations for technology acceptance. *J Am Med Inform Assoc*. 2016;23(1):157-161.
38. Stanley E, Cradock A, Bisset J, McEntee C, O'Connell MJ. Impact of sensory design interventions on image quality, patient anxiety and overall patient experience at MRI. *Br J Radiol.* 2016;89(1067).
39. Prakash B. Patient satisfaction. *J Cutan Aesthet Surg.* 2010;3(3):151-155.
40. Austin Z, Sutton J. Qualitative research: getting started. *Can J Hosp Pharm*. 2014;67(6):436-440.

For peer review only

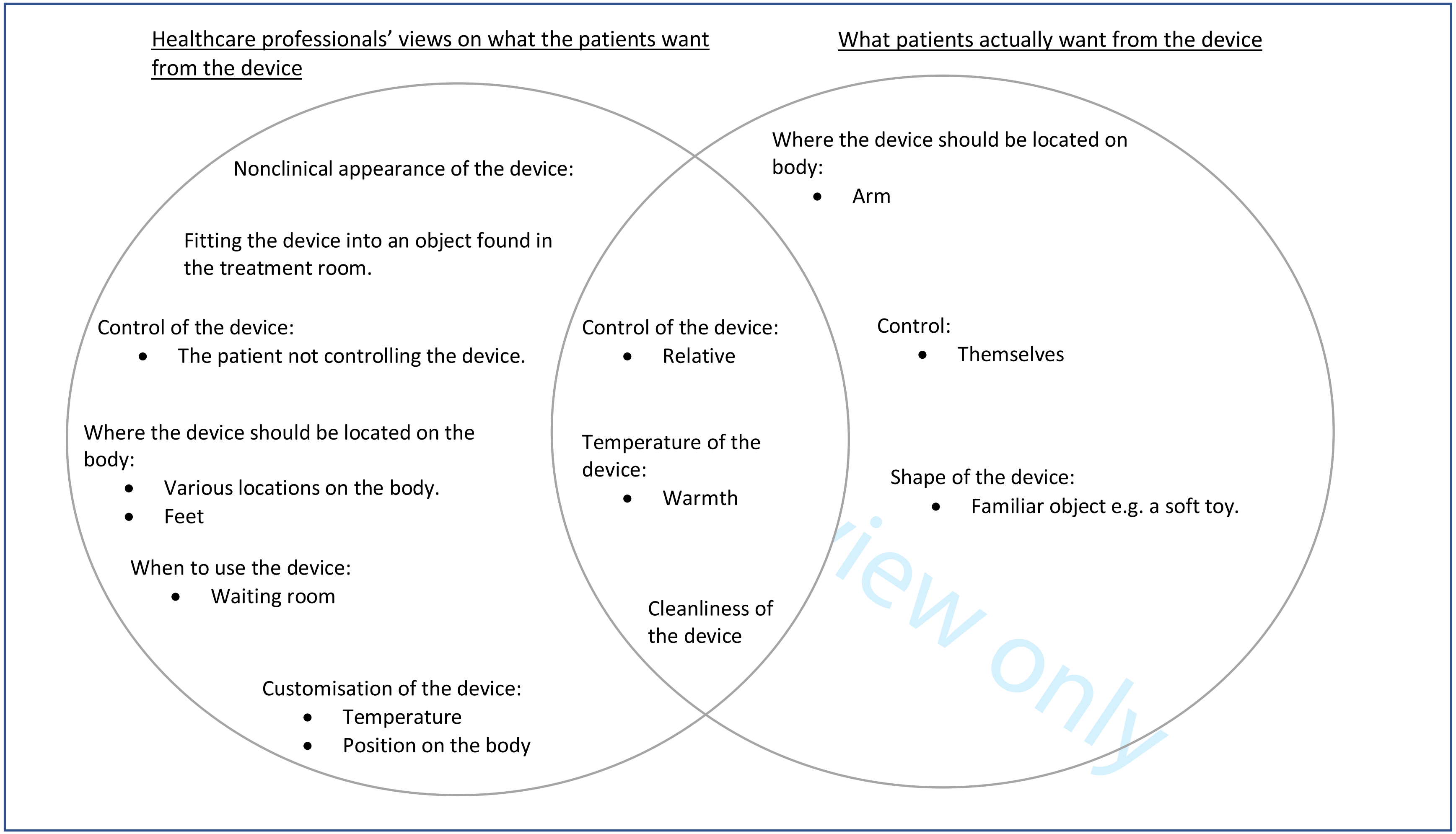
Healthcare professionals' views on what the patients want from the device
What patients actually want from the device
Nonclinical appearance of the device:
Fitting the device into an object found in the treatment room.
Control of the device:
• The patient not controlling the device.
Where the device should be located on the body:
• Various locations on the body.
• Feet
When to use the device:
• Waiting room
Customisation of the device:
• Temperature
• Position on the body
Control of the device:
• Relative
Temperature of the device:
• Warmth
Cleanliness of the device
Where the device should be located on body:
• Arm
Control:
• Themselves
Shape of the device:
• Familiar object e.g. a soft toy.

Standards for Reporting Qualitative Research (SRQR) as it applies to:

PATIENT AND PRACTITIONER PERSPECTIVES ON THE DESIGN OF A SIMULATED AFFECTIVE TOUCH DEVICE TO REDUCE PROCEDURAL ANXIETY ASSOCIATED WITH RADIOTHERAPY: A QUALITATIVE STUDY

O'Brien B.C., Harris, I.B., Beckman, T.J., Reed, D.A., & Cook, D.A. (2014). Standards for reporting qualitative research: a synthesis of recommendations. *Academic Medicine, 89(9)*, 1245-1251.

**No. Topic**	**Location in Article (page number)**
**Title and abstract**	N.B. Page numbers are based off the proof page numbers.
S1 Title	Page 2
S2 Abstract	Page 3
**Introduction**	
S3 Problem formulation	Page 5
S4 Purpose or research question	Page 6
**Methods**	
S5 Qualitative approach and research paradigm	Page 6
S6 Researcher characteristics and reflexivity	Pages 8
S7 Context	Page 7
S8 Sampling strategy	Page 7
S9 Ethical issues pertaining to human subjects	Page 6
S10 Data collection methods	Page 7
S11 Data collection instruments and technologies	Page 7
S12 Units of study	Page 7-8
S13 Data processing	Pages 8
S14 Data analysis	Page 8-9
S15 Techniques to enhance trustworthiness	Page 8-9
**Results/Findings**	
S16 Synthesis and interpretation	Pages 9-13
S17 Links to empirical data	Pages 9-13
**Discussion**	

For peer review only

S18 Integration with prior work, implications, transferability, and contribution(s) to the field	Pages 13-16
S19 Limitations	Page 15
**Other**	
S20 Conflicts of interest	Page 2
S21 Funding	Page 2

For peer review only

PATIENT AND PRACTITIONER PERSPECTIVES ON THE DESIGN OF A SIMULATED AFFECTIVE TOUCH DEVICE TO REDUCE PROCEDURAL ANXIETY ASSOCIATED WITH RADIOTHERAPY: A QUALITATIVE STUDY

Journal:	*BMJ Open*
Manuscript ID	bmjopen-2021-050288.R2
Article Type:	Original research
Date Submitted by the Author:	27-Nov-2021
Complete List of Authors:	Hall, Hugo ; King's College London; Imperial College London<br>Dhuga, Yasmin; Brighton and Sussex Medical School; Imperial College London<br>Yan Zheng, Caroline; Royal College of Art<br>Clunie, Gemma; Imperial College London Department of Surgery and Cancer, Sackler MSK Lab; Imperial College Healthcare NHS Trust, Speech and Language Therapy<br>Joyce, Elizabeth; Royal Marsden NHS Foundation Trust<br>McNair, Helen; Institute of Cancer Research, Radiotherapy and Imaging ; Royal Marsden NHS Foundation Trust<br>McGregor, Alison; Imperial College London, Dept of Surgery and Cancer
**Primary Subject Heading**:	Radiology and imaging
Secondary Subject Heading:	Oncology, Qualitative research
Keywords:	RADIOTHERAPY, QUALITATIVE RESEARCH, ONCOLOGY

SCHOLARONE™ Manuscripts

For peer review only - http://bmjopen.bmj.com/site/about/guidelines.xhtml

I, the Submitting Author has the right to grant and does grant on behalf of all authors of the Work (as defined in the below author licence), an exclusive licence and/or a non-exclusive licence for contributions from authors who are: i) UK Crown employees; ii) where BMJ has agreed a CC-BY licence shall apply, and/or iii) in accordance with the terms applicable for US Federal Government officers or employees acting as part of their official duties; on a worldwide, perpetual, irrevocable, royalty-free basis to BMJ Publishing Group Ltd ("BMJ") its licensees and where the relevant Journal is co-owned by BMJ to the co-owners of the Journal, to publish the Work in this journal and any other BMJ products and to exploit all rights, as set out in our licence.

The Submitting Author accepts and understands that any supply made under these terms is made by BMJ to the Submitting Author unless you are acting as an employee on behalf of your employer or a postgraduate student of an affiliated institution which is paying any applicable article publishing charge ("APC") for Open Access articles. Where the Submitting Author wishes to make the Work available on an Open Access basis (and intends to pay the relevant APC), the terms of reuse of such Open Access shall be governed by a Creative Commons licence – details of these licences and which Creative Commons licence will apply to this Work are set out in our licence referred to above.

Other than as permitted in any relevant BMJ Author's Self Archiving Policies, I confirm this Work has not been accepted for publication elsewhere, is not being considered for publication elsewhere and does not duplicate material already published. I confirm all authors consent to publication of this Work and authorise the granting of this licence.

For peer review only

PATIENT AND PRACTITIONER PERSPECTIVES ON THE DESIGN OF A SIMULATED AFFECTIVE TOUCH DEVICE TO REDUCE PROCEDURAL ANXIETY ASSOCIATED WITH RADIOTHERAPY: A QUALITATIVE STUDY

Corresponding/senior authors:

Alison McGregor (a.mcgregor@imperial.ac.uk), Helen McNair (Helen.McNair@rmh.nhs.uk)

Co-authors:

Hugo Hall, Yasmin Dhuga, Caroline Yan Zheng, Gemma Clunie, Elizabeth Joyce, Helen McNair, Alison McGregor.

Hugo Hall and Yasmin Dhuga contributed equally to this paper.

Word count: 4703
Abstract – 226

Funding statement:

This research project was funded by the CRUK Convergence Science Centre at The Institute of Cancer Research, London, and Imperial College London (A26234)

We also acknowledge NHS funding to the NIHR Biomedical Research Centre at The Royal Marsden and The Institute of Cancer Research. The views expressed in this publication are those of the authors and not necessarily those of those of the NHS or the National Institute for Health Research.

Helen McNair is funded by a National Institute for Health Research and Health Education England (HEE/NIHR), Senior Clinical Lecturer award (ICA-SCL-2018-04-ST2-002).

Gemma Clunie is funded by the National Institute for Health Research (NIHR) Imperial Biomedical Research Centre and the NIHR Clinical Doctoral Research Fellowship Programme CDRF-2017-03-028/ Integrated Clinical Academic Programme.

Alison McGregor acknowledges support from the NIHR Imperial Biomedical Research Centre (BRC).

Disclosure Statement: No potential conflict of interest is reported by the authors.

Data Statement: Data (focus group transcripts) is held at Royal Marsden NHS Foundation Trust and can be accessed on request by contacting: Helen.McNair@rmh.nhs.uk

For peer review only

ABSTRACT

Objective The aim of this study was to elicit the views of relevant stakeholders on the design of a device using simulated affective touch to reduce procedural anxiety surrounding radiotherapy and imaging.

Design This qualitative study collected data from focus groups which were then analysed using inductive thematic analysis in line with Braun and Clarke's methods.

Participants and setting Twenty patients and carers were recruited, as well as ten healthcare practitioners involved in either delivering radiotherapy or imaging procedures.

Results Patients, carers and healthcare practitioners agreed on some aspects of the device design, such as ensuring the device is warm and flexible in where it can be used on the body. However, patient and healthcare practitioner cohorts had at times differing viewpoints. For example, healthcare practitioners provided professional perspectives and required easy cleaning of the device. Meanwhile patients focused on anxiety relieving factors, such as the tactile sensation of the device being either a vibration or pulsation. There was no consensus on who should control the device.

Conclusions The desired features of a simulated affective touch device have been investigated. Different priorities of patients, carers and healthcare practitioners were evident. Any design must incorporate such features as to appease both groups. Areas where no consensus was reached could be further explored, alongside including further patient and public involvement in the form of a project advisory group.

Key words: qualitative, focus groups, radiotherapy, procedural anxiety, tactile sensation

For peer review only

ARTICLE SUMMARY

STRENGTHS AND LIMITATIONS OF THIS STUDY

Strength This is the first qualitative study to explore whether a tactile intervention provides a useful tool in reducing procedural anxiety for radiotherapy patients.

Strength This study explores the opinions of two separate stakeholder cohorts, each with different viewpoints (personal and professional) around the design of the device.

Limitation All participants were selected from a single geographical area which may indicate a homogenous focus group composition.

Limitation Our study cannot be generalised to all individuals who receive radiotherapy, as this study only considered adult participants.

For peer review only

PATIENT AND PRACTITIONER PERSPECTIVES ON THE DESIGN OF A SIMULATED AFFECTIVE TOUCH DEVICE TO REDUCE PROCEDURAL ANXIETY ASSOCIATED WITH RADIOTHERAPY: A QUALITATIVE STUDY

INTRODUCTION

Currently 458,000 people live with cancer in the UK (1). Over 50% of these patients will be managed with radiotherapy (2, 3), which can cause procedural anxiety, with approximately 49% of patients experiencing anxiety and psychological distress (4, 5). Procedural anxiety refers to excessive worry or fear of medical procedures (6) and is a phenomenon exacerbated with new developments in radiotherapy, for example stereotactic and adaptive radiotherapy, that require long treatment times.

High levels of distress during radiotherapy can directly impact the accuracy and efficacy of the procedure (7). However, the consequences of procedural anxiety extend beyond radiotherapy and include diagnostic imaging, and other invasive procedures performed on the conscious patient. Between 2-5% of magnetic resonance imaging (MRI) scans are terminated due to procedural anxiety (8, 9); this equates to a significant financial cost for the NHS considering that 3.4 million MRI scans were conducted between 2017 to 2018 (10).

Pre-existing interventions to manage procedural anxiety are limited and there is a need for an appropriate anxiety alleviating strategy or device in medical settings (11-13). Techniques such as verbal communication or music may distract the patient but do not address the reported sense of isolation associated with separation from caregivers or families. Sedation is commonly used to tackle procedural anxiety in the context of MRI but has implications for cost, risk and time (9). This has implications in the context of daily radiotherapy treatment. On top of increased risk to the patient, sedation requires the presence of an anaesthetics team which has time implications for the treatment, facility and hospital resources.

Research indicates that human affective (or empathetic) touch 'provides both psychological and physical comfort' Peled-Avron et al (14). However, relatives and carers are prohibited from being in the radiotherapy treatment room, leaving patients alone. Since the COVID-19 pandemic, patients are even more isolated with many hospitals not allowing visitors into waiting areas. As such, this research focuses on patient's perspectives of a simulated affective touch (SAT) device - a haptic device that uses tactile simulations to mimic attributes of human affective touch.

Haptic devices simulate attributes of human affective touch, such as gentle stroking, which has been shown to produce pleasant sensation (15), relieve psychological distress (16) and reduce the sense of social isolation associated with being apart from caregivers (17-19). However, such tactile interventions have not been widely explored as a tool to reduce procedural anxiety in radiotherapy patients. It is postulated that SAT can provide a non-invasive intervention to combat procedural anxiety surrounding radiotherapy due to their ability to reproduce natural touch (20).

For peer review only - http://bmjopen.bmj.com/site/about/guidelines.xhtml

The following paper aims to investigate patient, carer and healthcare professional (HCP) views on the creation of a calming device using SAT to combat induced procedural anxiety. Patients were asked about what aspects of any SAT device they would find calming. Carers play a vital role in supporting their loved ones through difficult times, a view endorsed by a recent study MacMillan survey (21) which reported 74% of carers supported their loved ones by talking and listening to them. Therefore, we can assume they have insight on the impact of medical procedures on their loved ones and also have a role in the requirements of such a device. Finally, the views of HCPs as key stakeholders in any potential device were collected to ensure future clinical usability and translation. HCP views were collected in order to advise on professional requirements (e.g. cleanability), as well as what they thought patients might appreciate about any calming SAT device. We intend to use the insights of the above groups to facilitate the future development of a design brief for an SAT prototype device to reduce procedural anxiety. This will ensure that the prototype device will be relevant and suitable for future clinical settings.

METHODS

Study Design

This was a qualitative co-design study using focus groups to elicit the views of two distinct categories of participants (first patients and carers, secondly HCPs), on the creation of an SAT device for use during radiotherapy interventions.

The purpose of the focus group was to establish participant thoughts around the experience of four soft robotic tactile intervention probing artefacts, which all had different shapes, touch patterns and interaction options. First, we established the ground rules and agenda of the focus group. Then the four probing artefacts were introduced to the group to serve as prompts. This enabled participants to engage in discussion around the desirable and undesirable features of a tactile intervention device.

Example questions included:

A) How participants feel about the sensations
B) Whether they would consider the device useful in helping to alleviate possible anxiety during the care journey
C) Where during the care journey they think this device would be useful
D) What sort of sensations they liked and would prefer

We adopted a phenomenological approach using inductive thematic analysis. This allowed us to focus on how best to use the product design to counter the phenomena of procedural anxiety around radiotherapy and imaging.

The study protocol was approved by Greater Manchester South Research Ethics Committee, and permission to conduct the research was granted by Health and Care Research Wales. REC reference: 19/NW/0607, Protocol number: CCR5133, IRAS project ID: 266016.

For peer review only - http://bmjopen.bmj.com/site/about/guidelines.xhtml

Participants and Setting

Participants were recruited from two groups of stakeholders: one comprised both patients and carers, the other group comprised HCPs. Patients and carers were invited to participate as experts on the lived experience of having radiotherapy and/or imaging procedures; this allowed us to explore circumstances and situations that may have created anxiety and to provide ideas on how to manage these. The views of HCPs involved in delivering radiotherapy or imaging procedures were important as key stakeholders if the device is to become part of clinical practice.

Convenience sampling was used to select both participant groups. Inclusion criteria stipulated participants were aged over 18 years, while those patients who were considered too unwell (by self-report or in consultation with clinical staff) were excluded. Those who were unable to understand English without an interpreter were also excluded. Patients and their carers were contacted using flyers placed in radiotherapy treatment and pre-treatment waiting rooms, while HCPs were internally recruited by departmental email. Patients from the local Biomedical Research Centre (BRC) Patient and Public Involvement (PPI) groups were also emailed. Information sheets were distributed to interested patients and carers who then gave written informed consent to join the study. Written informed consent was also obtained from HCPs participating in the study.

Data Collection

A total of six focus groups took place at the Royal Marsden Hospital. The total number of participants was 30 (table 1), with four patient and caregiver groups (two groups with four participants, two groups with six participants) and two HCP groups (five participants each). Of the 10 HCPs, nine were therapeutic radiographers (eight involved in the planning or treatment of radiotherapy patients and one educational lead) and one was a diagnostic radiographer working mainly in magnetic resonance imaging. The patient and caregiver focus groups lasted on average 83 minutes (ranging from 82 – 85 minutes). The HCP focus groups lasted on average 49 minutes (ranging from 48 – 50 minutes). Recruitment was stopped after six focus groups when initial analysis of the themes demonstrated no new emerging themes, indicative of saturation (22).

For peer review only

	Patient had experience of radiotherapy	Cared for someone with radiotherapy experience	Other*	Worked as a healthcare practitioner in radiotherapy
**Totals**	13	3	4	10
**Gender**				
**Male**	4	1	3	-
**Female**	9	2	1	10
**Age**				
**18-25**	-	-		1
**26-35**	-	-		5
**36-45**	-	-		2
**46-55**	2	-		2
**56-65**	7	1	2	-
**66-75**	3	2	2	-
**76-85**	1	-		-

Focus groups were recorded and transcribed verbatim for analysis. Each focus group was conducted by two facilitators with qualitative cancer research experience and were supported by the design researcher. All qualitative researchers involved in leading the focus groups had no prior relationship with the patient participants but two of them were well known to the HCP cohort. The varied comments and depth of discussion implies that that there was sufficient freedom for articulating thoughts in each of the groups. The design researcher was not known to any participants.

Data Analysis

Thematic analysis was used to identify themes and sub-themes inductively within the focus group data. In line with Braun and Clarke's (23) methods, the initial step was for researchers to familiarise themselves with the interview recordings. Next, transcripts were analysed using QSR NVivo Release 1.1 and all information relating to calming measures was coded. Each transcript was double coded (once by the design researcher and once by the medical researchers) allowing for multiple perspectives on the analysis. An independent researcher then reviewed the codes to increase inter-coder reliability and the trustworthiness of the results (24). Periodic meetings about the analysis with the whole project team led to iterations and finally the collation of the codes into themes and sub-themes.

Patient and Public Involvement

The concept was presented at initial planning stages to two Royal Marsden NHS Patient and Public (PPI) groups. Positive feedback was received which enabled us to proceed with study.

RESULTS

Three overarching themes emerged from analysis of the data: patient and carer views on what they want from the device, HCP views on the device and HCP views on what they believe patients want from the device.

Further analysis of the data identified seven key sub-themes based on what patients wanted from the device; control of the device, temperature of the device, cleanliness of the device, where the device should be located on the body, shape of the device, visual appearance of the device and tactile sensation of the device.

In addition, two key sub-themes were identified based on what HCPs would want from the device from a professional point of view: functionality of the device and universal compatibility of the device.

Finally, there were eight key sub-themes based on what HCPs believe patients would like; non-clinical appearance of the device, fitting the device into an object found in the treatment room, who controls the device, where the device should be located on the body, cleanliness of the device, temperature of the device, when to use the device and customisation of the device. These themes are summarised in figure 1 and discussed in more detail below.

[Figure 1 near here]

PATIENT AND CARER VIEWS

Control of the device:
Patients and carers reached strong consensus in favour of being able to control the device themselves. They highlighted that in times of uncertainty, the device would give them something to be in control of. Another popular opinion included a relative being able to control the device from home as many liked the idea of being able to interact with a loved one at a distance.

"That will make a big difference. And you've no control in these situations, so I think if you being able to control it probably would be quite reassuring."
-P7: Cared for someone with radiotherapy experience

Temperature of the device:

For peer review only

The most discussed feature of the device, and the most important feature for some patients and carers, included the temperature of the device. The consensus was that the device should provide the patient with warmth.

"It's also temperature, when you hold someone's hand, it's a warm experience, whereas sometimes with some of the treatments you have, you feel quite cold and distant, and that's a bit of a strange one. It's like saying to someone it was all warm and fuzzy, it's a stupid thing to say, but actually temperature and touch and a feeling of wellbeing all go together, really."
-P17: Patient had experience of radiotherapy

Cleanliness of the device:
Only one patient expressed a desire for a clean device and stated that they would not want to use a device shaped like a glove if someone else had used it.

"…but then hygiene wise. If someone has used the glove, I wouldn't want to use it afterwards. But that's me."
-P7: Cared for someone with radiotherapy experience

Where the device should be located on the body:
Patient and carer preferences for location on the body included a wide variety of locations: the hand, arm, shoulder, neck, head, leg, foot, buttocks, whole body and customisable – being able to use the same device in multiple locations. However, the most popular answer was using the device around the arm either because of a medical reason or personal preference.

"Maybe, for me, the arms, because I've got lymphedema, and that can be annoying."
-P10: Other (imaging but not radiotherapy experience)

Shape of the device:
Patients and carers expressed interest in the shape of the device. The ball shaped probing artifact received good feedback with several participants expressing positive sentiments towards it based on its shape. Other participants suggested the shape of a teddy bear or comforting soft toy to help make associations with happier times.

"the ball, it would just give you a degree of confidence as someone who isn't so secure in where they are."
-P19: Patient had experience of radiotherapy

Visual appearance of the device:
Patients and carers discussed making the device visually appealing so that it looked more interesting.

"Would it be possible to put lights or something inside it, so that it comes on… where the bits come out, colourful as well? "
-P12: Patient had experience of radiotherapy

For peer review only - http://bmjopen.bmj.com/site/about/guidelines.xhtml

Tactile sensation of the device:
Overall, patients and carers liked the idea of the device having a distinct tactile sensation, whether it was a pulsation, ripple, tapping, or vibration. These motions were described as comforting and a form of distraction.

"It's clever isn't it? If you get it in the right pulse, it's just so calming. It is the ripple up and the ripple down, modulating."
-P3: Patient had experience of radiotherapy

HEALTHCARE PRACTITIONER VIEWS

Non-clinical appearance of the device:
All HCPs expressed a negative attitude towards the device appearing like a piece of clinical equipment. This was due to their belief that this would be off-putting to the patient. Two HCPS expressed that they felt blue was the correct colour for the device from a personal perspective.

"you wouldn't want it to look too clinical"
-P27: radiographer

"because of the patient age group. They might think, oh, you're taking my blood pressure."
-P29: radiographer

Fitting the device into an object found in the treatment room:
The HCPs believed that patients would favour a device that was pre-fitted into an object found in the treatment room. They believed this would benefit patients who were nervous about disclosing their anxiety. From a professional point of view, the HCPs also believed that a piece of equipment designed to be left in-situ would be more efficient.

"You don't actually have to ask for something. You don't even have to say yes, I'd like something for my anxiety."
-P23: radiographer

Control of the device:
The HCPs had different ideas about who the patient would most appreciate being in control of the device. Some HCPs believed that family members controlling the device would be the most comforting; enabling the patient to control the device could introduce a new anxiety on top of radiotherapy. Additionally, from a professional point of view, HCPs were concerned about giving patients control of the device. They were worried that the device could distract patients and affect their ability to remain still for the radiotherapy treatment or imaging.

"Yes, definitely that element of someone else can control it is great. And I think, actually, a lot of patients would find that really comforting to know that their family, I don't know, their children, or something, was doing that I think."
-P26: radiographer

For peer review only

“I just wonder if [controlling the device] introduces too many elements... They're overwhelmed already...He’s relaxed, then he switches it off, then in treatment... They then panic because they want it back on. I think it introduces too many overwhelming factors.”
-P28: radiographer

Where the device should be located on the body:
One of the most widely held views among HCPs was that patients would want a flexible device that could be used in various locations on the body. If the device was not adaptable in terms of location, the second most favourable solution was to design a device for use on the feet. This was suggested both because of its similarity to a foot massage as well as because this is a location that was judged to be compatible with many radiotherapy set-ups.

“Maybe the ability for it to go... Adaptability, maybe, of different parts of the body or different equipment. Adaptability.”
-P28: radiographer

Cleanliness of the device:
In contrast to patients, HCP were keen to have a device that was easily cleanable and wipeable, with one group listing it as their top feature required when considering the practicality of an SAT device for use in radiotherapy. This research was conducted pre-COVID-19 pandemic, and so this feature would likely be of even greater importance now that infection control procedures and the disinfection of all equipment post-use have been brought into such sharp focus.

“Everything would need to be wipeable.”
-P27: radiographer

“it has to pass infection control”
-P25: radiographer

“Facilitator: Practicalities. Where would you fit the practicalities?
P27: I would say that it’s cleanable being up at the top.
P28: It has to be top, yes...”
-P27, P28: both radiographers

Temperature of the device:
In a point of similarity to the patients and carers, most HCPs believed that a warm device would be calming to a patient. However, a few HCPs disagreed and suggested that it would be good to adjust the temperature based on the needs of the patient.

“And, for me, I always like to make sure they’re warm. Our treatment rooms can be quite cold. And if somebody is anxious, I don’t know, I always think if I’m cold I’ll be more anxious. So, it might be getting them an extra blanket, or making sure they’ve got an extra gown or something.”
-P25: radiographer

For peer review only

When to use the device:
Some HCPs believed patients would want to use the device in the waiting room to alleviate anxiety.

"And that's I think one of the first points where you can really tackle that anxiety as such and alleviate some of it."
-P28: radiographer

Customisation of the device:
Overall, there was agreement on the fact that the device should be customisable in various ways in order to meet everyone's preferences. For example, patients should be able to choose whether they want to be able to feel the warmth, the tactile sensation employed and the position on the body the device is used.

DISCUSSION

Receiving cancer treatment can be a stress-provoking experience for patients for many reasons, including separation from their loved ones, an issue that is currently very pertinent (4). With recent advances in radiotherapy, patients are required to spend an increasing amount of time in the treatment room, which may exacerbate such problems. Anxious patients may be managed pharmacologically but this is not appropriate for all patients and can be time consuming. On the non-pharmacological side, there are no universally used interventions in radiotherapy departments, and research into new interventions is limited (25). Further, any non-pharmacological interventions currently being investigated do not make up for the lack of physical interaction experienced by patients, instead focusing on distracting patients (for example through music).

We have identified key design aspects for an SAT device that may alleviate the anxiety patients experience during radiotherapy and/or imaging. The implementation of such a device may help reduce costs through a smaller number of cancelled procedures as well as less spent on medication to combat procedural anxiety.

Overall, we found that participant cohorts had mixed opinions on the design aspects of such a device. As suggested by Crowe et al. (26), this could have been because of their different roles (HCP versus patient/carer) and, therefore, different priorities.

Perhaps unsurprisingly, HCPs were concerned about the device not disrupting their workflow. For example, they emphasised designing an easily cleanable device, this is consistent with the literature which suggests interruptions to their workload may contribute to medical errors (27).

Control of the device was a controversial topic. Patients expressed the desire to have control of the device, whereas HCPs were keen for patients not to have control. HCP feared that patient control would lead to disruptions to the treatment session. Both groups agreed

For peer review only

that allowing relatives some degree of control of the device would be good. This finding is supported by other studies that have shown the involvement of friends and family in radiotherapy treatment has a positive impact on patient care (28). This controversy on control of the device warrants further exploration in future research. Specifically, how useful a device to reduce anxiety would be if it was controlled by the HCP against the patient's preference – relative control may provide a happy middle ground in this regard.

Future research should also incorporate further patient and public involvement and engagement, perhaps in the form of a project advisory group. Involving such an advisory group at all stages of the research could help to make sure the patient perspective is not neglected (29), ensuring any device is both amenable to patients as well as HCPs.

HCPs were divided regarding when the device should be used, with some suggesting patients might appreciate a calming device in the waiting room. Other HCPs suggested the treatment room, which conforms with patient views. The varying views are reflected in the literature which suggests that there is not a particular time that is anxiety-provoking for patients, and that anxiety can start a few days before the procedure and end days after (30, 31). Clarification on where exactly anxiety is most often experienced would be an ideal topic for future qualitative focus group studies.

If designed around the treatment room, HCPs suggest the device be left in-situ attached to the radiotherapy couch. The easy accessibility may benefit some patients who are anxious but do not report it. Patients from Black, Asian, and Minority Ethnic (BAME) communities are less likely to disclose and seek help for mental health issues but are at higher risk of mental health disorders. Reasons for not disclosing this information include either not recognising the symptoms or being in denial of mental health issues as a result of cultural beliefs (32, 33). The Department of Health has recognised the need to close the healthcare gap for BAME populations (31). Leaving the device in-situ would help address procedural anxiety for those who are against disclosing that they are anxious.

Patients appreciated the simulated affective touch aspect of the device. Vibration and pulsation sensations were valued for their relaxing comfort and distraction, whereas 'stroking' motions were described as too human to be performed by a machine. The idea that simulated affective touch has the potential to relieve psychological distress is supported by the literature (16). However, it may be important not to make it too realistic as to fall into the hypothesised 'uncanny valley' (34) of imperfectly representing human features and actions.

Designing a device that is warm to touch was the idea most universally supported by both participant cohorts. Heat therapy has been shown to reduce the amount of serum cortisol and noradrenaline levels, hence downregulating the over-activated sympathetic nervous system in anxiety (35). Moreover, studies on various populations have demonstrated that heat therapy is an effective anxiolytic intervention in medical and non-medical situations (11, 12, 35).

HCPs emphasised the importance of a non-clinical visual appearance, particularly if applying the device to the arm. They were concerned patients may associate it with a blood pressure

For peer review only - http://bmjopen.bmj.com/site/about/guidelines.xhtml

cuff, a device that can induce spike anxiety in certain patients (36). On the other hand, the only patient requirement was to make the device look visually appealing using lights and colour. Aesthetic factors have been suggested as important considerations in the healthcare setting because they may influence whether patients will use the device (37).

HCPs often gave their views on device design from a professional standpoint, for example, by suggesting a design which can be applied to multiple different areas of the body. This would allow personalisation of the device so patients can use the device wherever they feel might be most comforting. Radiotherapy for head and neck cancer can be particularly anxiety-inducing due to the need for a close-fitting mask (13), and it may be prudent to make sure any device design accommodates these patients.

If this device proves to be beneficial for patients undergoing radiotherapy, it may have the potential to be used in other clinical settings to alleviate anxiety. MRI scans are known to cause anxiety (38), and there have been investigations into interventions prior to the procedure to alleviate anxiety. Interventions have included calls to or videos for patients to watch which explain the procedure (13). Whilst reducing anxiety levels, these interventions cannot be delivered in-situ during the procedure (13), which is something that an SAT device could achieve.

More generally, the literature suggests that there is a need for an appropriate anxiety alleviating device in the medical setting (11-13) but a solution has not previously been found. The case for a remote-controlled SAT device operated by a relative, has applicability to the COVID-19 pandemic where imposed social distancing (maintaining a 2-meter distance from others) and many hospitals prohibiting in-person visitors lead to high levels of distress amongst patients and their families.

There are several limitations to this study. The use of convenience sampling may have resulted in a selection bias, leading to more homogenous focus groups with under- or over-representation of particular cohorts. For example, all participants were selected from a single geographical area. Aside from sampling limitations, the collection of demographic data could have provided useful insight into the impact of ethnicity on design preferences for this device. The study also only considered adult participants; future studies may consider how the design of an SAT device might differ for children. Finally, a limitation of focus groups is that participants may not feel able or want to share their views, or that one or two people may dominate the discussion. We attempted to address these limitations by creating a relaxed environment and using experienced moderators.

The fact that this study uses two separate participant cohorts with different viewpoints (personal and professional) is a strength. Patient opinions are vital to any intervention & meeting patient needs will enhance satisfaction and therefore improve the overall clinical outcome (39). However, patient desires need to be balanced with HCP requirements as if the device was designed in a way that disrupted the treatment it would be unlikely to be implemented. Further qualitative research could be conducted on this topic with the use of a patient and public involvement project advisory group at all stages of the study to make sure the interests of both groups are equally represented (29, 40).

For peer review only

CONCLUSION

In conclusion, we have identified there is a lack of data around the benefit of using tactile interventions as a tool to reduce procedural anxiety in radiotherapy patients. This study identified which design features of an anxiety reducing tactile intervention would be valued by HCPs, patients and their carers. HCPs and patients agree on some features: a warm device which is flexible in where it can be used on the body (hence accommodating patients no matter where they are receiving radiotherapy). Both agreed that relatives should have some control over the device. HCPs wanted to ensure the device can easily be cleaned while patients were keen for the vibration and pulsation sensations to be used to put them at ease. Areas where no consensus was reached could be further explored as well as including greater patient and public involvement in the form of a project advisory group.

The authors intend to incorporate the desirable design features identified from this study to develop a design brief for a soft robotic device to alleviate procedural anxiety. The next stage would be to create a prototype device to test the usability and utility of this concept.

For peer review only

For peer review only - http://bmjopen.bmj.com/site/about/guidelines.xhtml

FIGURE/TABLE LEGENDS

Table 1. Description of focus groups and participants. A total of 30 participants were recruited, 13 radiotherapy patients, 10 healthcare practitioners and three carers. Four other participants had not received radiotherapy or cared for someone who had experienced radiotherapy. 'Other' refers to cancer patients recruited from the BRC Patient and public involvement group *who had received imaging (e.g. MRI) but not radiotherapy.*

Figure 1- An outline of the overarching themes and the subsequent subthemes within. The Venn diagram demonstrates where the patients and HCPs agree and disagree with each other.

AUTHORS CONTRIBUTIONS

Hugo Hall and Yasmin Dhuga contributed equally to the composition of this paper as well as the thematic analysis of the data and wish to be credited as co-lead authors if possible.

Alison McGregor and Helen McNair were the senior authors on this project and provided directorial oversight.

Helen McNair, Alison McGregor and Caroline Yan Zheng developed the study design and focus group guide.

Caroline Yan Zheng and Elizabeth Joyce and Gemma Clunie facilitated the focus groups alongside Helen McNair.

Caroline also conducted initial data analysis and, alongside Gemma Clunie, provided feedback on draft manuscript versions.

For peer review only

REFERENCES

1. International Agency for Research on Cancer. Cancer tomorrow - Estimated number of new cases from 2020 to 2040. https://gco.iarc.fr/tomorrow/en/dataviz/isotype [accessed 15th December 2020].
2. Delaney G, Jacob S, Featherstone C, Barton M. The role of radiotherapy in cancer treatment: estimating optimal utilization from a review of evidence-based clinical guidelines. *Cancer* 2005;104(6): 1129-37.
3. Begg AC, Stewart FA, Vens C. Strategies to improve radiotherapy with targeted drugs. *Nat Rev Cancer*. 2011;11(4):239-53.
4. Elsner K, Naehrig D, Halkett GKB, Dhillon HM. Reduced patient anxiety as a result of radiation therapist-led psychosocial support: a systematic review. *J Med Radiat Sci.* 2017;64(3):220-31.
5. Holmes N, Williamson K. A survey of cancer patients undergoing a radical course of radiotherapy, to establish levels of anxiety and depression. *J Radiother Pract.* 2008;7(2):89-98.
6. Choy Y. Acute procedure anxiety in adults: course, screening, assessment, and differential diagnosis: UpToDate, 2019. Available: https://www.uptodate.com/contents/acute-procedure-anxiety-in-adults-course-screening-assessment-and-differential-diagnosis [Accessed 18 Mar 2019].
7. Royal College of Radiologists (2008) *On Target: Ensuring Geometric Accuracy in Radiotherapy*; p. 78. https://www.rcr.ac.uk/system/files/publication/field_publication_files/BFCO%2808%295_On_target.pdf [accessed 12 December 2020].
8. Chapman HA, Bernier D, Rusak B. MRI-related anxiety levels change within and between repeated scanning sessions. *Psychiatry Res.* 2010;182(2):160-4.
9. Murphy KJ, Brunberg JA. Adult claustrophobia, anxiety and sedation in MRI. *Magn Reson Imaging.* 1997;15(1):51-4.
10. National Health Service (2019) Diagnostic Imaging Dataset Annual Statistical Release 2018/2019. https://www.england.nhs.uk/statistics/wp-content/uploads/sites/2/2019/12/Annual-Statistical-Release-2018-19-PDF-1.9MB.pdf [accessed 21 December 2020].
11. Kim HJ, Kim JW, Park HS, Moon DG, Lee JG, Oh MM. The use of a heating pad to reduce anxiety, pain, and distress during cystoscopy in female patients. *Int Urogynecol J.* 2019;30(10):1705-10.
12. Hedén L, Von Essen L, Ljungman G. Randomized interventions for needle procedures in children with cancer. *Eur J Cancer Care (Engl).*2009;18(4):358-63.
13. Tugwell JR, Goulden N, Mullins P. Alleviating anxiety in patients prior to MRI: A pilot single-centre single-blinded randomised controlled trial to compare video demonstration or telephone conversation with a radiographer versus routine intervention. *Radiography (Lond).* 2018;24(2):122-9.
14. Peled-Avron L, Levy-Gigi E, Richter-Levin G, Korem N, Shamay-Tsoory SG. The role of empathy in the neural responses to observed human social touch. *Cognit Affect Behav Neurosci*. 2016;16(5):802–813.
15. Zheng, C. Y. et al. (2021) 'Comparing soft robotic affective touch to human and brush affective touch', in *2021 IEEE World Haptics Conference (WHC)*. [Online]. July 2021 pp. 352–352.

For peer review only - http://bmjopen.bmj.com/site/about/guidelines.xhtml

16. Triscoli C, Croy I, Steudte-Schmiedgen S, Olausson H, Sailer U. Heart rate variability is enhanced by long-lasting pleasant touch at CT-optimized velocity. *Biol Psychol*. 2017;128:71-81.
17. Parmar R, Brewer BB, Szalacha LA. Foot Massage, Touch, and Presence in Decreasing Anxiety during a Magnetic Resonance Imaging: A Feasibility Study. *J Altern Complement Med*. 2018;24(3):268-75.
18. Kolcaba K, Schirm V, Steiner R. Effects of hand massage on comfort of nursing home residents. *Geriatr Nurs*. 2006;27(2):85-91.
19. Von Mohr M, Kirsch LP, Fotopoulou A. The soothing function of touch: affective touch reduces feelings of social exclusion. *Sci Rep*. 2017;7(1):13516.
20. Goldsworthy, S. et al. The potential for haptic touch technology to supplement human empathetic touch during radiotherapy. Journal of Medical Imaging and Radiation Sciences. [Online]. 2020; 51 (4, Supplement); S39–S43.
21. Macmillan Cancer Support. Understanding the numbers, needs and experiences of cancer carers in the UK. https://www.macmillan.org.uk/_images/carers-of-people-with-cancer_tcm9-282780.pdf [accessed 9th September 2021].
22. Faulkner SL, Trotter SP. Data Saturation. The International Encyclopedia of Communication Research Methods. 2017:1-2.
23. Braun V, Clarke V. Using thematic analysis in psychology. *Qual Res Psychol*. 2006;3(2):77-101.
24. Castleberry A, Nolen A. Thematic analysis of qualitative research data: Is it as easy as it sounds? *Curr Pharm Teach Learn*. 2018;10(6):807-815.
25. Forbes E, Baker AL, Britton B, Clover K, Skelton E, Oultram S, et al. Non-pharmacological approaches to procedural anxiety reduction for patients undergoing radiotherapy for cancer: systematic review protocol. *BMJ Open*. 2020;10(10):e035155.
26. Crowe S, Fenton M, Hall M, Cowan K, Chalmers I. Patients', clinicians' and the research communities' priorities for treatment research: there is an important mismatch. *Res Involv Engagem*. 2015;1(1):2.
27. Rivera-Rodriguez AJ, Karsh BT. Interruptions and distractions in healthcare: review and reappraisal. *Qual Saf Health Care*. 2010;19(4):304-12.
28. Mackenzie LJ, Sanson-Fisher RW, Carey ML, D'Este CA. Radiation oncology outpatient perceptions of patient-centred care: a cross-sectional survey. *BMJ Open*. 2013;3(2).
29. NIHR Involve. Briefing note three: Why involve members of the public in research? [Internet] https://www.invo.org.uk/posttyperesource/why-should-members-of-the-public-be-involved-in-research/ [accessed 24 January 2021].
30. Appukuttan DP. Strategies to manage patients with dental anxiety and dental phobia: literature review. *Clin Cosmet Investig Dent*. 2016;8:35-50.
31. Szczepura A. Access to health care for ethnic minority populations. *PMJ*. 2005;81(953):141.
32. Memon A, Taylor K, Mohebati LM, Sundin J, Cooper M, Scanlon T, et al. Perceived barriers to accessing mental health services among black and minority ethnic (BME) communities: a qualitative study in Southeast England. *BMJ Open*. 2016;6(11).
33. Weich S, Nazroo J, Sproston K, McManus S, Blanchard M, Erens B, et al. Common mental disorders and ethnicity in England: the EMPIRIC study. Psychol Med. 2004;34(8):1543-1551.
34. Mathur MB, Reichling DB. Navigating a social world with robot partners: A quantitative cartography of the Uncanny Valley. *Cognition*. 2016;146:22-32.

For peer review only

35. Lee YH, Park BN, Kim SH. The effects of heat and massage application on autonomic nervous system. *Yonsei Med J.* 2011;52(6):982-989.
36. Dillon KM, Seacat JD, Saucier CD, Doyle-Campbell CJ. Could Blood Pressure Phobia Go Beyond the White Coat Effect? *Am J Hypertens*. 2015;28(11):1306-1309.
37. Lazard AJ, Watkins I, Mackert MS, Xie B, Stephens KK, Shalev H. Design simplicity influences patient portal use: the role of aesthetic evaluations for technology acceptance. *J Am Med Inform Assoc*. 2016;23(1):157-161.
38. Stanley E, Cradock A, Bisset J, McEntee C, O'Connell MJ. Impact of sensory design interventions on image quality, patient anxiety and overall patient experience at MRI. *Br J Radiol.* 2016;89(1067).
39. Prakash B. Patient satisfaction. *J Cutan Aesthet Surg.* 2010;3(3):151-155.
40. Austin Z, Sutton J. Qualitative research: getting started. *Can J Hosp Pharm*. 2014;67(6):436-440.

For peer review only

Figure 1- An outline of the overarching themes and the subsequent subthemes within. The Venn diagram demonstrates where the patients and HCPs agree and disagree with each other.

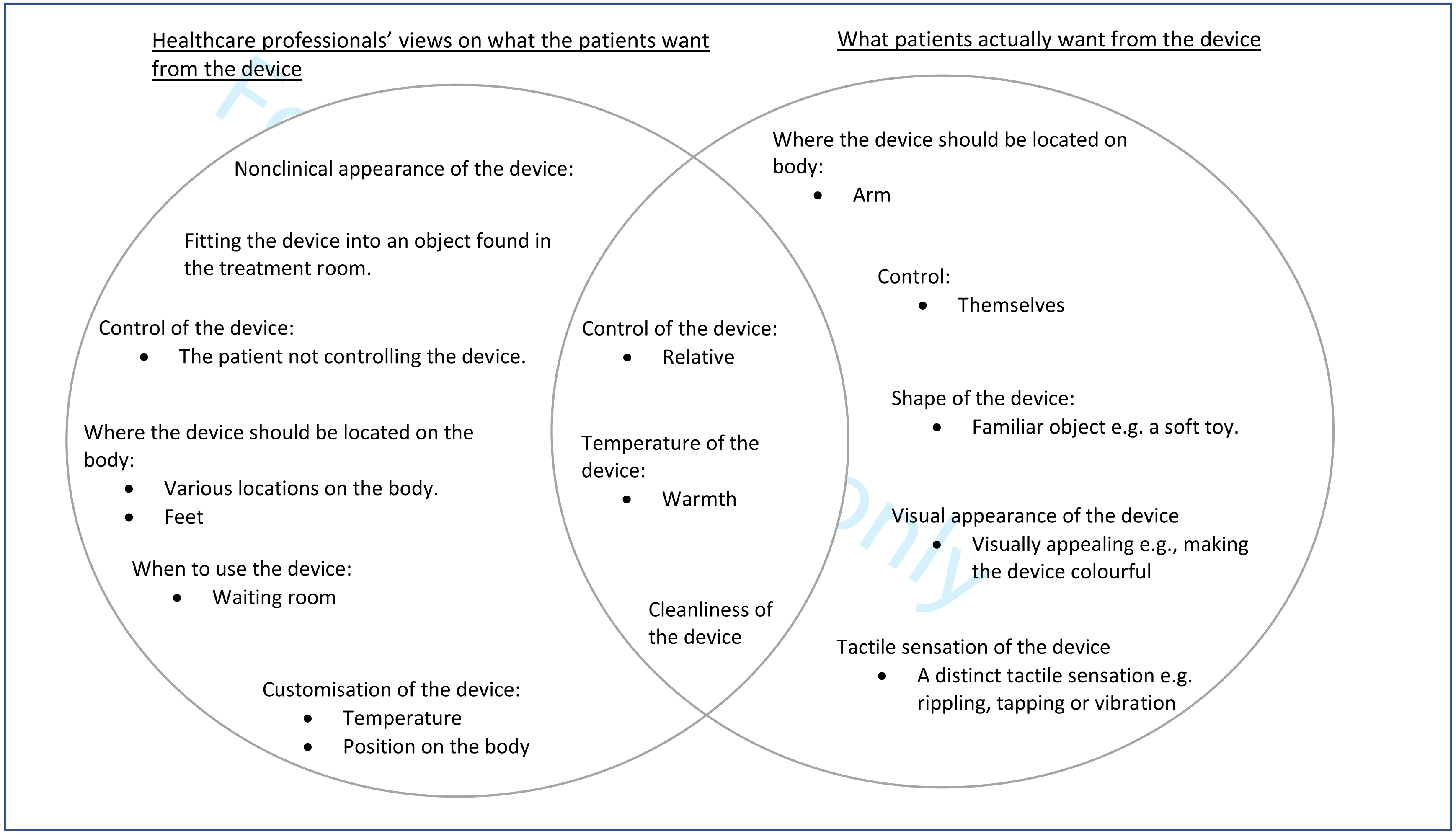

Standards for Reporting Qualitative Research (SRQR) as it applies to:

PATIENT AND PRACTITIONER PERSPECTIVES ON THE DESIGN OF A SIMULATED AFFECTIVE TOUCH DEVICE TO REDUCE PROCEDURAL ANXIETY ASSOCIATED WITH RADIOTHERAPY: A QUALITATIVE STUDY

O'Brien B.C., Harris, I.B., Beckman, T.J., Reed, D.A., & Cook, D.A. (2014). Standards for reporting qualitative research: a synthesis of recommendations. *Academic Medicine, 89(9)*, 1245-1251.

For peer review only

No. Topic	Location in Article (page number)
**Title and abstract**	N.B. Page numbers are based off the proof page numbers.
S1 Title	Page 2
S2 Abstract	Page 3
**Introduction**	
S3 Problem formulation	Page 5
S4 Purpose or research question	Page 6
**Methods**	
S5 Qualitative approach and research paradigm	Page 6
S6 Researcher characteristics and reflexivity	Page 8
S7 Context	Page 7
S8 Sampling strategy	Page 7
S9 Ethical issues pertaining to human subjects	Page 6
S10 Data collection methods	Page 7
S11 Data collection instruments and technologies	Page 7
S12 Units of study	Page 7-8
S13 Data processing	Pages 8
S14 Data analysis	Page 8
S15 Techniques to enhance trustworthiness	Page 8
**Results/Findings**	
S16 Synthesis and interpretation	Pages 9-13
S17 Links to empirical data	Pages 9-13
**Discussion**	

S18 Integration with prior work, implications, transferability, and contribution(s) to the field	Pages 13-15
S19 Limitations	Page 15
**Other**	
S20 Conflicts of interest	Page 2
S21 Funding	Page 2

For peer review only

BMJ Open

PATIENT AND PRACTITIONER PERSPECTIVES ON THE DESIGN OF A SIMULATED AFFECTIVE TOUCH DEVICE TO REDUCE PROCEDURAL ANXIETY ASSOCIATED WITH RADIOTHERAPY: A QUALITATIVE STUDY

Journal:	*BMJ Open*
Manuscript ID	bmjopen-2021-050288.R3
Article Type:	Original research
Date Submitted by the Author:	23-Feb-2022
Complete List of Authors:	Hall, Hugo ; King's College London; Imperial College London<br>Dhuga, Yasmin; Brighton and Sussex Medical School; Imperial College London<br>Yan Zheng, Caroline; Royal College of Art<br>Clunie, Gemma; Imperial College London Department of Surgery and Cancer, Sackler MSK Lab; Imperial College Healthcare NHS Trust, Speech and Language Therapy<br>Joyce, Elizabeth; Royal Marsden NHS Foundation Trust<br>McNair, Helen; Institute of Cancer Research, Radiotherapy and Imaging ; Royal Marsden NHS Foundation Trust<br>McGregor, Alison; Imperial College London, Dept of Surgery and Cancer
**Primary Subject Heading**:	Radiology and imaging
Secondary Subject Heading:	Oncology, Qualitative research
Keywords:	RADIOTHERAPY, QUALITATIVE RESEARCH, ONCOLOGY

SCHOLARONE™
Manuscripts

I, the Submitting Author has the right to grant and does grant on behalf of all authors of the Work (as defined in the below author licence), an exclusive licence and/or a non-exclusive licence for contributions from authors who are: i) UK Crown employees; ii) where BMJ has agreed a CC-BY licence shall apply, and/or iii) in accordance with the terms applicable for US Federal Government officers or employees acting as part of their official duties; on a worldwide, perpetual, irrevocable, royalty-free basis to BMJ Publishing Group Ltd ("BMJ") its licensees and where the relevant Journal is co-owned by BMJ to the co-owners of the Journal, to publish the Work in this journal and any other BMJ products and to exploit all rights, as set out in our licence.

The Submitting Author accepts and understands that any supply made under these terms is made by BMJ to the Submitting Author unless you are acting as an employee on behalf of your employer or a postgraduate student of an affiliated institution which is paying any applicable article publishing charge ("APC") for Open Access articles. Where the Submitting Author wishes to make the Work available on an Open Access basis (and intends to pay the relevant APC), the terms of reuse of such Open Access shall be governed by a Creative Commons licence – details of these licences and which Creative Commons licence will apply to this Work are set out in our licence referred to above.

Other than as permitted in any relevant BMJ Author's Self Archiving Policies, I confirm this Work has not been accepted for publication elsewhere, is not being considered for publication elsewhere and does not duplicate material already published. I confirm all authors consent to publication of this Work and authorise the granting of this licence.

For peer review only

PATIENT AND PRACTITIONER PERSPECTIVES ON THE DESIGN OF A SIMULATED AFFECTIVE TOUCH DEVICE TO REDUCE PROCEDURAL ANXIETY ASSOCIATED WITH RADIOTHERAPY: A QUALITATIVE STUDY

Corresponding/senior authors:

Alison H McGregor (a.mcgregor@imperial.ac.uk), Helen A McNair (Helen.McNair@rmh.nhs.uk)

Co-authors:

Hugo Hall, Yasmin Dhuga, Caroline Yan Zheng, Gemma Clunie, Elizabeth Joyce, Helen McNair, Alison McGregor.

Hugo Hall and Yasmin Dhuga contributed equally to this paper.

Word count: 4599
Abstract – 228

Funding statement:

This research project was funded by the CRUK Convergence Science Centre at The Institute of Cancer Research, London, and Imperial College London (A26234)

We also acknowledge NHS funding to the NIHR Biomedical Research Centre at The Royal Marsden and The Institute of Cancer Research. The views expressed in this publication are those of the authors and not necessarily those of those of the NHS or the National Institute for Health Research.

Helen McNair is funded by a National Institute for Health Research and Health Education England (HEE/NIHR), Senior Clinical Lecturer award (ICA-SCL-2018-04-ST2-002).

Gemma Clunie is funded by the National Institute for Health Research (NIHR) Imperial Biomedical Research Centre and the NIHR Clinical Doctoral Research Fellowship Programme CDRF-2017-03-028/ Integrated Clinical Academic Programme.

Alison McGregor acknowledges support from the NIHR Imperial Biomedical Research Centre (BRC).

Disclosure Statement: No potential conflict of interest is reported by the authors.

Data Statement: Data (focus group transcripts) is held at Royal Marsden NHS Foundation Trust and can be accessed on request by contacting: Helen.McNair@rmh.nhs.uk

For peer review only

ABSTRACT

Objective The aim of this study was to elicit the views of relevant stakeholders on the design of a device using simulated affective touch to reduce procedural anxiety surrounding radiotherapy and imaging.

Design This qualitative study collected data from focus groups which were then analysed using inductive thematic analysis in line with Braun and Clarke's methods.

Participants and setting Twenty patients and carers were recruited, as well as ten healthcare practitioners involved in either delivering radiotherapy or imaging procedures.

Results Patients, carers and healthcare practitioners agreed on some aspects of the device design, such as ensuring the device is warm and flexible in where it can be used on the body. However, patient and healthcare practitioner cohorts had at times differing viewpoints. For example, healthcare practitioners provided professional perspectives and required easy cleaning of the device. Meanwhile patients focused on anxiety relieving factors, such as the tactile sensation of the device being either a vibration or pulsation. There was no consensus on who should control the device.

Conclusions The desired features of a simulated affective touch device have been investigated. Different priorities of patients and their carers and healthcare practitioners were evident. Any design must incorporate such features as to appease both groups. Areas where no consensus was reached could be further explored, alongside including further patient and public involvement in the form of a project advisory group.

Key words: qualitative, focus groups, radiotherapy, procedural anxiety, tactile sensation

For peer review only

ARTICLE SUMMARY

STRENGTHS AND LIMITATIONS OF THIS STUDY

Strength This is the first qualitative study to explore whether a tactile intervention provides a useful tool in reducing procedural anxiety for radiotherapy patients.

Strength This study explores the opinions of two separate stakeholder cohorts, each with different viewpoints (personal and professional) around the design of the device.

Limitation All participants were selected from a single geographical area which may indicate a homogenous focus group composition.

Limitation Our study cannot be generalised to all individuals who receive radiotherapy, as this study only considered adult participants.

For peer review only

For peer review only - http://bmjopen.bmj.com/site/about/guidelines.xhtml

PATIENT AND PRACTITIONER PERSPECTIVES ON THE DESIGN OF A SIMULATED AFFECTIVE TOUCH DEVICE TO REDUCE PROCEDURAL ANXIETY ASSOCIATED WITH RADIOTHERAPY: A QUALITATIVE STUDY

INTRODUCTION

Currently 458,000 people live with cancer in the UK (1). Over 50% of these patients will be managed with radiotherapy (2, 3), which can cause procedural anxiety, with approximately 49% of patients experiencing anxiety and psychological distress (4, 5). Procedural anxiety refers to excessive worry or fear of medical procedures (6) and is a phenomenon exacerbated with new developments in radiotherapy, for example stereotactic and adaptive radiotherapy, that require long treatment times.

High levels of distress during radiotherapy can directly impact the accuracy and efficacy of the procedure (7). Procedural anxiety is not limited to radiotherapy and also occurs during diagnostic imaging, and other invasive procedures performed on a conscious patient. Between 2-5% of magnetic resonance imaging (MRI) scans are terminated due to procedural anxiety (8, 9); this equates to a significant financial cost for the NHS considering that 3.4 million MRI scans were conducted between 2017 to 2018 (10).

Pre-existing interventions to manage procedural anxiety are limited and there is a need for an appropriate anxiety alleviating strategy or device in medical settings (11-13). Techniques such as verbal communication or music may distract the patient but do not address the reported sense of isolation associated with separation from caregivers or families. Sedation is commonly used to tackle procedural anxiety in the context of MRI but has implications for cost, risk, and time (9). Sedation especially if treatment is required on a daily basis has implications on service provision, facility usage and hospital resoures, and repeated sedation may have associated medical risks in some patient groups.

Research indicates that human affective (or empathetic) touch 'provides both psychological and physical comfort' Peled-Avron et al (14). However, relatives and carers are prohibited from being in the radiotherapy treatment room, leaving patients alone. Since the COVID-19 pandemic, patients are even more isolated with many hospitals not allowing visitors into waiting areas. As such, this research focuses on patient's perspectives of a simulated affective touch (SAT) device - a haptic device that uses tactile simulations to mimic attributes of human affective touch.

Haptic devices simulate attributes of human affective touch, such as gentle stroking, which has been shown to produce pleasant sensation (15), relieve psychological distress (16) and reduce the sense of social isolation associated with being apart from caregivers (17-19). However, such tactile interventions have not been widely explored as a tool to reduce procedural anxiety in radiotherapy patients. It is postulated that SAT can provide a non-invasive intervention to combat procedural anxiety surrounding radiotherapy due to their ability to reproduce natural touch (20).

For peer review only - http://bmjopen.bmj.com/site/about/guidelines.xhtml

The following paper aims to investigate patient, carer and healthcare professional (HCP) views on the creation of a calming device using SAT to combat induced procedural anxiety. Patients were asked about what aspects of any SAT device they would find calming. Carers play a vital role in supporting their loved ones through difficult times, a view endorsed by a recent study MacMillan survey (21) which reported 74% of carers supported their loved ones by talking and listening to them. Therefore, we can assume they have insight on the impact of medical procedures on their loved ones and also have a role in the requirements of such a device. Finally, the views of HCPs as key stakeholders in any potential device were collected to ensure future clinical usability and translation. HCP views were collected in order to advise on professional requirements (e.g. cleanability), as well as what they thought patients might appreciate about any calming SAT device. We intend to use the insights of the above groups to facilitate the future development of a design brief for an SAT prototype device to reduce procedural anxiety. This will ensure that the prototype device will be relevant and suitable for future clinical settings.

METHODS

Study Design

This was a qualitative co-design study using focus groups to elicit the views of two distinct categories of participants (first patients and carers, secondly HCPs), on the creation of an SAT device for use during radiotherapy interventions.

The purpose of the focus group was to establish participant thoughts around the experience of four soft robotic tactile intervention probing artefacts, which all had different shapes, touch patterns and interaction options. First, we established the ground rules and agenda of the focus group. Then the four probing artefacts were introduced to the group to serve as prompts. This enabled participants to engage in discussion around the desirable and undesirable features of a tactile intervention device.

Example questions included:

A) How participants feel about the sensations
B) Whether they would consider the device useful in helping to alleviate possible anxiety during the care journey
C) Where during the care journey they think this device would be useful
D) What sort of sensations they liked and would prefer

We adopted a phenomenological approach using inductive thematic analysis. This allowed us to focus on how best to use the product design to counter the phenomena of procedural anxiety around radiotherapy and imaging.

The study protocol was approved by Greater Manchester South Research Ethics Committee, and permission to conduct the research was granted by Health and Care Research Wales. REC reference: 19/NW/0607, Protocol number: CCR5133, IRAS project ID: 266016.

For peer review only - http://bmjopen.bmj.com/site/about/guidelines.xhtml

Participants and Setting

Participants were recruited from two groups of stakeholders: one comprised both patients and carers, the other group comprised HCPs. Patients and carers were invited to participate as experts on the lived experience of having radiotherapy and/or imaging procedures; this allowed us to explore circumstances and situations that may have created anxiety and to provide ideas on how to manage these. The views of HCPs involved in delivering radiotherapy or imaging procedures were important as key stakeholders if the device is to become part of clinical practice.

Convenience sampling was used to select both participant groups. Inclusion criteria stipulated participants were aged over 18 years, while those patients who were considered too unwell (by self-report or in consultation with clinical staff) were excluded. Those who were unable to understand English without an interpreter were also excluded. Patients and their carers were contacted using flyers placed in radiotherapy treatment and pre-treatment waiting rooms, while HCPs were internally recruited by departmental email. Patients from the local Biomedical Research Centre (BRC) Patient and Public Involvement (PPI) groups were also emailed. Information sheets were distributed to interested patients and carers who then gave written informed consent to join the study. Written informed consent was also obtained from HCPs participating in the study.

Data Collection

A total of six focus groups took place at the Royal Marsden Hospital. The total number of participants was 30 (table 1), with four patient and caregiver groups (two groups with four participants, two groups with six participants) and two HCP groups (five participants each). Of the 10 HCPs, nine were therapeutic radiographers (eight involved in the planning or treatment of radiotherapy patients and one educational lead) and one was a diagnostic radiographer working mainly in magnetic resonance imaging. The patient and caregiver focus groups lasted on average 83 minutes (ranging from 82 – 85 minutes). The HCP focus groups lasted on average 49 minutes (ranging from 48 – 50 minutes). Recruitment was stopped after six focus groups when initial analysis of the themes demonstrated no new emerging themes, indicative of saturation (22).

For peer review only

	Patient had experience of radiotherapy	Cared for someone with radiotherapy experience	Other*	Worked as a healthcare practitioner in radiotherapy
**Totals**	13	3	4	10
**Gender**				
**Male**	4	1	3	-
**Female**	9	2	1	10
**Age**				
**18-25**	-	-		1
**26-35**	-	-		5
**36-45**	-	-		2
**46-55**	2	-		2
**56-65**	7	1	2	-
**66-75**	3	2	2	-
**76-85**	1	-		-

Table 1. Description of focus groups and participants. A total of 30 participants were recruited, 13 radiotherapy patients, 10 healthcare practitioners and three carers. Four other participants had not received radiotherapy or cared for someone who had experienced radiotherapy.
**'Other' refers to cancer patients recruited from the* BRC Patient and public involvement group *who had received imaging (e.g. MRI) but not radiotherapy.*

Focus groups were recorded and transcribed verbatim for analysis. Each focus group was conducted by two medical facilitators with qualitative cancer research experience and were supported by the design researcher. All qualitative researchers involved in leading the focus groups had no prior relationship with the patient participants but two of them were well known to the HCP cohort. The varied comments and depth of discussion implies that that there was sufficient freedom for articulating thoughts in each of the groups. The design researcher was not known to any participants.

Data Analysis

Thematic analysis was used to identify themes and sub-themes inductively within the focus group data. In line with Braun and Clarke's (23) methods, the initial step was for researchers to familiarise themselves with the interview recordings. Next, transcripts were analysed using QSR NVivo Release 1.1 and all information relating to calming measures was coded. Each transcript was double coded (once by the design researcher and once by the medical

For peer review only

researchers) allowing for multiple perspectives on the analysis. An independent researcher then reviewed the codes to increase inter-coder reliability and the trustworthiness of the results (24). Periodic meetings about the analysis with the whole project team led to iterations and finally the collation of the codes into themes and sub-themes.

Patient and Public Involvement

The concept was presented at initial planning stages to two Royal Marsden NHS Patient and Public (PPI) groups. Positive feedback was received which enabled us to proceed with study.

RESULTS

Three overarching themes emerged from analysis of the data: patient and carer views on what they want from the device, HCP views on the device and HCP views on what they believe patients want from the device.

Further analysis of the data identified seven key sub-themes based on what patients wanted from the device; control of the device, temperature of the device, cleanliness of the device, where the device should be located on the body, shape of the device, visual appearance of the device and tactile sensation of the device.

There were eight key sub-themes based on what HCPs believe patients would like; non-clinical appearance of the device, fitting the device into an object found in the treatment room, who controls the device, where the device should be located on the body, cleanliness of the device, temperature of the device, when to use the device and customisation of the device. These themes are summarised in figure 1 and discussed in more detail below.

[Figure 1 near here]

PATIENT AND CARER VIEWS

Control of the device:
Patients and carers reached strong consensus in favour of the patient being able to control the device themselves. They highlighted that in times of uncertainty, the device would give the patient something to be in control of. Another popular opinion included a relative being able to control the device from home as many liked the idea of being able to interact with a loved one at a distance.

"That will make a big difference. And you've no control in these situations, so I think if you being able to control it probably would be quite reassuring."
-P7: Cared for someone with radiotherapy experience

Temperature of the device:

For peer review only - http://bmjopen.bmj.com/site/about/guidelines.xhtml

The most discussed feature of the device, and the most important feature for some patients and carers, included the temperature of the device. The consensus was that the device should provide the patient with warmth.

"It's also temperature, when you hold someone's hand, it's a warm experience, whereas sometimes with some of the treatments you have, you feel quite cold and distant, and that's a bit of a strange one. It's like saying to someone it was all warm and fuzzy, it's a stupid thing to say, but actually temperature and touch and a feeling of wellbeing all go together, really."
-P17: Patient had experience of radiotherapy

Cleanliness of the device:
Only one patient expressed a desire for a clean device and stated that they would not want to use a device shaped like a glove if someone else had used it.

"…but then hygiene wise. If someone has used the glove, I wouldn't want to use it afterwards. But that's me."
-P7: Cared for someone with radiotherapy experience

Where the device should be located on the body:
Patient and carer preferences for location on the body included a wide variety of locations: the hand, arm, shoulder, neck, head, leg, foot, buttocks, whole body and customisable – being able to use the same device in multiple locations. However, the most popular answer was using the device around the arm either because of a medical reason or personal preference.

"Maybe, for me, the arms, because I've got lymphedema, and that can be annoying."
-P10: Other (imaging but not radiotherapy experience)

Shape of the device:
Patients and carers expressed interest in the shape of the device. The ball shaped probing artefact received good feedback with several participants expressing positive sentiments towards it based on its shape. Other participants suggested the shape of a teddy bear or comforting soft toy to help make associations with happier times.

"the ball, it would just give you a degree of confidence as someone who isn't so secure in where they are."
-P19: Patient had experience of radiotherapy

Visual appearance of the device:
Patients and carers discussed making the device visually appealing so that it looked more interesting.

"Would it be possible to put lights or something inside it, so that it comes on… where the bits come out, colourful as well? "
-P12: Patient had experience of radiotherapy

For peer review only - http://bmjopen.bmj.com/site/about/guidelines.xhtml

Tactile sensation of the device:
Overall, patients and carers liked the idea of the device having a distinct tactile sensation, whether it was a pulsation, ripple, tapping, or vibration. These motions were described as comforting and a form of distraction.

"It's clever isn't it? If you get it in the right pulse, it's just so calming. It is the ripple up and the ripple down, modulating."
-P3: Patient had experience of radiotherapy

HEALTHCARE PRACTITIONER VIEWS

Non-clinical appearance of the device:
All HCPs expressed a negative attitude towards the device appearing like a piece of clinical equipment. This was due to their belief that this would be off-putting to the patient. Two HCPS expressed that they felt blue was the correct colour for the device from a personal perspective.

"you wouldn't want it to look too clinical"
-P27: radiographer

"because of the patient age group. They might think, oh, you're taking my blood pressure."
-P29: radiographer

Fitting the device into an object found in the treatment room:
The HCPs believed that patients would favour a device that was pre-fitted into an object found in the treatment room. They believed this would benefit patients who were nervous about disclosing their anxiety. From a professional point of view, the HCPs also believed that a piece of equipment designed to be left in-situ would be more efficient.

"You don't actually have to ask for something. You don't even have to say yes, I'd like something for my anxiety."
-P23: radiographer

Control of the device:
The HCPs had different ideas about who the patient would most appreciate being in control of the device. Some HCPs believed that family members controlling the device would be the most comforting; enabling the patient to control the device could introduce a new anxiety on top of radiotherapy. Additionally, from a professional point of view, HCPs were concerned about giving patients control of the device. They were worried that the device could distract patients and affect their ability to remain still for the radiotherapy treatment or imaging.

"Yes, definitely that element of someone else can control it is great. And I think, actually, a lot of patients would find that really comforting to know that their family, I don't know, their children, or something, was doing that I think."
-P26: radiographer

For peer review only

"I just wonder if [controlling the device] introduces too many elements... They're overwhelmed already...He's relaxed, then he switches it off, then in treatment... They then panic because they want it back on. I think it introduces too many overwhelming factors."
-P28: radiographer

Where the device should be located on the body:
One of the most widely held views among HCPs was that patients would want a flexible device that could be used in various locations on the body. If the device was not adaptable in terms of location, the second most favourable solution was to design a device for use on the feet. This was suggested both because of its similarity to a foot massage as well as because this is a location that was judged to be compatible with many radiotherapy set-ups.

"Maybe the ability for it to go... Adaptability, maybe, of different parts of the body or different equipment. Adaptability."
-P28: radiographer

Cleanliness of the device:
HCP were keen to have a device that was easily cleanable and wipeable, with one group listing it as their top feature required when considering the practicality of an SAT device for use in radiotherapy. This research was conducted pre-COVID-19 pandemic, and so this feature would likely be of even greater importance now that infection control procedures and the disinfection of all equipment post-use have been brought into such sharp focus.

"Everything would need to be wipeable."
-P27: radiographer

"it has to pass infection control"
-P25: radiographer

"Facilitator: Practicalities. Where would you fit the practicalities?
P27: I would say that it's cleanable being up at the top.
P28: It has to be top, yes..."
-P27, P28: both radiographers

Temperature of the device:
In a point of similarity to the patients and carers, most HCPs believed that a warm device would be calming to a patient. However, a few HCPs disagreed and suggested that it would be good to adjust the temperature based on the needs of the patient.

"And, for me, I always like to make sure they're warm. Our treatment rooms can be quite cold. And if somebody is anxious, I don't know, I always think if I'm cold I'll be more anxious. So, it might be getting them an extra blanket, or making sure they've got an extra gown or something."
-P25: radiographer
When to use the device:

For peer review only

Some HCPs believed patients would want to use the device in the waiting room to alleviate anxiety.

"And that's I think one of the first points where you can really tackle that anxiety as such and alleviate some of it."
-P28: radiographer

Customisation of the device:
Overall, there was agreement on the fact that the device should be customisable in various ways in order to meet everyone's preferences. For example, patients should be able to choose whether they want to be able to feel the warmth, the tactile sensation employed and the position on the body the device is used.

DISCUSSION

Receiving cancer treatment can be a anxiety-provoking experience for patients for many reasons, including separation from their loved ones, an issue that is currently very pertinent (4). With recent advances in radiotherapy, patients are required to spend an increasing amount of time in the treatment room, which may exacerbate such problems. Anxious patients may be managed pharmacologically but this is not appropriate for all patients and can be time consuming. On the non-pharmacological side, there are no universally used interventions in radiotherapy departments, and research into new interventions is limited (25). Further, any non-pharmacological interventions currently being investigated do not address the lack of physical interaction experienced by patients, instead focusing on distracting patients (for example through music).

We have identified key design aspects for an SAT device that may alleviate the anxiety patients experience during radiotherapy and/or imaging. The implementation of such a device may help reduce costs through a smaller number of cancelled procedures as well as less spent on medication to combat procedural anxiety.

Overall, we found that participant cohorts had mixed opinions on the design aspects of such a device. This is not unexpected as HCP have different roles and priorities to the patient and their carer (26). Perhaps unsurprisingly, HCPs were concerned about the device not disrupting their workflow, for example, emphasis was placed on designing an easily cleanable device. This is consistent with the literature which suggests interruptions to their workload may contribute to medical errors (27).

Control of the device was a controversial topic. Patients expressed the desire to have control of the device, whereas HCPs were keen for patients not to have control. HCP feared that patient control would lead to disruptions to the treatment session. Both groups agreed that allowing relatives some degree of control of the device would be good. Future iterations of the device need to provide the level of control required by the patients whilst

For peer review only

addressing HCPs concerns in relation to service delivery. If the efficacy of the device is established this may facilitate greater acceptance by the HCPs.

Future research should also incorporate further patient and public involvement and engagement, perhaps in the form of a project advisory group. Involving such an advisory group at all stages of the research could help to make sure the patient perspective is not neglected (28), ensuring any device is both amenable to patients as well as HCPs.

HCPs were divided regarding when the device should be used, with some suggesting patients might appreciate a calming device in the waiting room. Other HCPs suggested the treatment room, which conforms with patient views. The varying views are reflected in the literature which suggests that there is not a particular time that is anxiety-provoking for patients, and that anxiety can start a few days before the procedure and end days after (29, 30). Clarification on where exactly anxiety is most often experienced would be an ideal topic for future qualitative focus group studies.

If designed around the treatment room, HCPs suggest the device be left in-situ attached to the radiotherapy couch. The easy accessibility may benefit some patients who are anxious but do not report it. For example, patients from Black, Asian, and Minority Ethnic (BAME) communities are less likely to disclose and seek help for mental health issues but are at higher risk of mental health disorders. Reasons for not disclosing this information include not recognising or being aware of the symptoms (31, 32). The Department of Health has recognised the need to close the healthcare gap for BAME populations (30). Leaving the device in-situ would help address procedural anxiety for those who are against disclosing that they are anxious.

Patients appreciated the simulated affective touch aspect of the device. Vibration and pulsation sensations were valued for their relaxing comfort and distraction, whereas 'stroking' motions were described as too human to be performed by a machine. The idea that simulated affective touch has the potential to relieve psychological distress is supported by the literature (16). However, it may be important not to make it too realistic as to fall into the hypothesised 'uncanny valley' (33) of imperfectly representing human features and actions.

Designing a device that is warm to touch was the idea most universally supported by both participant cohorts. Heat therapy has been shown to reduce the amount of serum cortisol and noradrenaline levels, hence downregulating the over-activated sympathetic nervous system in anxiety (34). Moreover, studies on various populations have demonstrated that heat therapy is an effective anxiolytic intervention in medical and non-medical situations (11, 12, 34).

HCPs emphasised the importance of a non-clinical visual appearance, particularly if applying the device to the arm. They were concerned patients may associate it with a blood pressure cuff, a device that can induce spike anxiety in certain patients (35). On the other hand, the only patient requirement was to make the device look visually appealing using lights and colour. Aesthetic factors have been suggested as important considerations in the healthcare setting because they may influence whether patients will use the device (36).

For peer review only - http://bmjopen.bmj.com/site/about/guidelines.xhtml

HCPs often gave their views on device design from a professional standpoint, for example, by suggesting a design which can be applied to multiple different areas of the body. This would allow personalisation of the device so patients can use the device wherever they feel might be most comforting. Radiotherapy for head and neck cancer can be particularly anxiety-inducing due to the need for a close-fitting mask (13), and it may be prudent to make sure any device design accommodates these patients.

If this device proves to be beneficial for patients undergoing radiotherapy, it may have the potential to be used in other clinical settings to alleviate anxiety. MRI scans are known to cause anxiety (37), and there have been investigations into interventions prior to the procedure to alleviate anxiety. Interventions have included phone calls to patients or videos for patients to watch which explain the procedure (13). These interventions cannot be delivered in-situ during the procedure (13), which is something that an SAT device could achieve, thereby enhancing its potential efficacy.

More generally, the literature suggests that there is a need for an appropriate anxiety alleviating device in the medical setting (11-13) but a solution has not previously been found. The case for a remote-controlled SAT device operated by a relative, has applicability to the COVID-19 pandemic where imposed social distancing (maintaining a 2-meter distance from others) and many hospitals prohibiting in-person visitors lead to high levels of distress amongst patients and their families.

There are several limitations to this study. The use of convenience sampling may have resulted in a selection bias, leading to more homogenous focus groups with under- or over-representation of particular cohorts. For example, all participants were selected from a single geographical area. Aside from sampling limitations, the collection of demographic data could have provided useful insight into the impact of ethnicity on design preferences for this device. The study also only considered adult participants; future studies may consider how the design of an SAT device might differ for children. Finally, a limitation of focus groups is that participants may not feel able or want to share their views, or that one or two people may dominate the discussion. We attempted to address these limitations by creating a relaxed environment and using experienced moderators.

A strength of this study is the use of two separate participant cohorts with different viewpoints (personal and professional). Patient opinions are vital to any intervention & meeting patient needs will enhance satisfaction and therefore improve the overall clinical outcome (38). However, for the device to be successful it needs to designed to meet the needs of the patient user and the requirements of minimal disruption to treatment delivery of the HCP, and as such further iterations require both patient and HCP involvement.

CONCLUSION

In conclusion, we have identified there is a lack of data around the benefit of using tactile interventions as a tool to reduce procedural anxiety in radiotherapy patients. This study identified which design features of an anxiety reducing tactile intervention would be valued by HCPs, patients and their carers. HCPs and patients agree on some features: a warm

For peer review only

device which is flexible in where it can be used on the body (hence accommodating patients no matter where they are receiving radiotherapy). Both agreed that relatives should have some control over the device. HCPs wanted to ensure the device can easily be cleaned while patients were keen for the vibration and pulsation sensations to be used to put them at ease. Areas where no consensus was reached could be further explored as well as including greater patient and public involvement in the form of a project advisory group.

The authors intend to incorporate the desirable design features identified from this study to develop a design brief for a soft robotic device to alleviate procedural anxiety. The next stage would be to create a prototype device to test the usability and utility of this concept.

For peer review only

FIGURE LEGENDS

Figure 1- An outline of the overarching themes and the subsequent subthemes within. The Venn diagram demonstrates where the patients and HCPs agree and disagree with each other.

AUTHORS CONTRIBUTIONS

Hugo Hall and Yasmin Dhuga contributed equally to the composition of this paper as well as the thematic analysis of the data and wish to be credited as co-lead authors if possible.

Alison McGregor and Helen McNair were the senior authors on this project and provided directorial oversight.

Helen McNair, Alison McGregor and Caroline Yan Zheng developed the study design and focus group guide.

Caroline Yan Zheng and Elizabeth Joyce and Gemma Clunie facilitated the focus groups alongside Helen McNair.

Caroline also conducted initial data analysis and, alongside Gemma Clunie, provided feedback on draft manuscript versions.

For peer review only

For peer review only - http://bmjopen.bmj.com/site/about/guidelines.xhtml

REFERENCES

1. International Agency for Research on Cancer. Cancer tomorrow - Estimated number of new cases from 2020 to 2040. https://gco.iarc.fr/tomorrow/en/dataviz/isotype [accessed 15th December 2020].
2. Delaney G, Jacob S, Featherstone C, Barton M. The role of radiotherapy in cancer treatment: estimating optimal utilization from a review of evidence-based clinical guidelines. *Cancer* 2005;104(6): 1129-37.
3. Begg AC, Stewart FA, Vens C. Strategies to improve radiotherapy with targeted drugs. *Nat Rev Cancer*. 2011;11(4):239-53.
4. Elsner K, Naehrig D, Halkett GKB, Dhillon HM. Reduced patient anxiety as a result of radiation therapist-led psychosocial support: a systematic review. *J Med Radiat Sci.* 2017;64(3):220-31.
5. Holmes N, Williamson K. A survey of cancer patients undergoing a radical course of radiotherapy, to establish levels of anxiety and depression. *J Radiother Pract.* 2008;7(2):89-98.
6. Choy Y. Acute procedure anxiety in adults: course, screening, assessment, and differential diagnosis: UpToDate, 2019. Available: https://www.uptodate.com/contents/acute-procedure-anxiety-in-adults-course-screening-assessment-and-differential-diagnosis [Accessed 18 Mar 2019].
7. Royal College of Radiologists (2008) *On Target: Ensuring Geometric Accuracy in Radiotherapy*; p. 78. https://www.rcr.ac.uk/system/files/publication/field_publication_files/BFCO%2808%295_On_target.pdf [accessed 12 December 2020].
8. Chapman HA, Bernier D, Rusak B. MRI-related anxiety levels change within and between repeated scanning sessions. *Psychiatry Res.* 2010;182(2):160-4.
9. Murphy KJ, Brunberg JA. Adult claustrophobia, anxiety and sedation in MRI. *Magn Reson Imaging.* 1997;15(1):51-4.
10. National Health Service (2019) Diagnostic Imaging Dataset Annual Statistical Release 2018/2019. https://www.england.nhs.uk/statistics/wp-content/uploads/sites/2/2019/12/Annual-Statistical-Release-2018-19-PDF-1.9MB.pdf [accessed 21 December 2020].
11. Kim HJ, Kim JW, Park HS, Moon DG, Lee JG, Oh MM. The use of a heating pad to reduce anxiety, pain, and distress during cystoscopy in female patients. *Int Urogynecol J.* 2019;30(10):1705-10.
12. Hedén L, Von Essen L, Ljungman G. Randomized interventions for needle procedures in children with cancer. *Eur J Cancer Care (Engl).*2009;18(4):358-63.
13. Tugwell JR, Goulden N, Mullins P. Alleviating anxiety in patients prior to MRI: A pilot single-centre single-blinded randomised controlled trial to compare video demonstration or telephone conversation with a radiographer versus routine intervention. *Radiography (Lond).* 2018;24(2):122-9.
14. Peled-Avron L, Levy-Gigi E, Richter-Levin G, Korem N, Shamay-Tsoory SG. The role of empathy in the neural responses to observed human social touch. *Cognit Affect Behav Neurosci*. 2016;16(5):802–813.
15. Zheng, C. Y. et al. (2021) ‘Comparing soft robotic affective touch to human and brush affective touch’, in *2021 IEEE World Haptics Conference (WHC)*. [Online]. July 2021 pp. 352–352.

For peer review only - http://bmjopen.bmj.com/site/about/guidelines.xhtml

16. Triscoli C, Croy I, Steudte-Schmiedgen S, Olausson H, Sailer U. Heart rate variability is enhanced by long-lasting pleasant touch at CT-optimized velocity. *Biol Psychol*. 2017;128:71-81.
17. Parmar R, Brewer BB, Szalacha LA. Foot Massage, Touch, and Presence in Decreasing Anxiety during a Magnetic Resonance Imaging: A Feasibility Study. *J Altern Complement Med*. 2018;24(3):268-75.
18. Kolcaba K, Schirm V, Steiner R. Effects of hand massage on comfort of nursing home residents. *Geriatr Nurs*. 2006;27(2):85-91.
19. Von Mohr M, Kirsch LP, Fotopoulou A. The soothing function of touch: affective touch reduces feelings of social exclusion. *Sci Rep*. 2017;7(1):13516.
20. Goldsworthy, S. et al. The potential for haptic touch technology to supplement human empathetic touch during radiotherapy. Journal of Medical Imaging and Radiation Sciences. [Online]. 2020; 51 (4, Supplement); S39–S43.
21. Macmillan Cancer Support. Understanding the numbers, needs and experiences of cancer carers in the UK. https://www.macmillan.org.uk/_images/carers-of-people-with-cancer_tcm9-282780.pdf [accessed 9th September 2021].
22. Faulkner SL, Trotter SP. Data Saturation. The International Encyclopedia of Communication Research Methods. 2017:1-2.
23. Braun V, Clarke V. Using thematic analysis in psychology. *Qual Res Psychol*. 2006;3(2):77-101.
24. Castleberry A, Nolen A. Thematic analysis of qualitative research data: Is it as easy as it sounds? *Curr Pharm Teach Learn*. 2018;10(6):807-815.
25. Forbes E, Baker AL, Britton B, Clover K, Skelton E, Oultram S, et al. Non-pharmacological approaches to procedural anxiety reduction for patients undergoing radiotherapy for cancer: systematic review protocol. *BMJ Open*. 2020;10(10):e035155.
26. Crowe S, Fenton M, Hall M, Cowan K, Chalmers I. Patients', clinicians' and the research communities' priorities for treatment research: there is an important mismatch. *Res Involv Engagem*. 2015;1(1):2.
27. Rivera-Rodriguez AJ, Karsh BT. Interruptions and distractions in healthcare: review and reappraisal. *Qual Saf Health Care*. 2010;19(4):304-12.
28. NIHR Involve. Briefing note three: Why involve members of the public in research? [Internet] https://www.invo.org.uk/posttyperesource/why-should-members-of-the-public-be-involved-in-research/ [accessed 24 January 2021].
29. Appukuttan DP. Strategies to manage patients with dental anxiety and dental phobia: literature review. *Clin Cosmet Investig Dent*. 2016;8:35-50.
30. Szczepura A. Access to health care for ethnic minority populations. *PMJ*. 2005;81(953):141.
31. Memon A, Taylor K, Mohebati LM, Sundin J, Cooper M, Scanlon T, et al. Perceived barriers to accessing mental health services among black and minority ethnic (BME) communities: a qualitative study in Southeast England. *BMJ Open*. 2016;6(11).
32. Weich S, Nazroo J, Sproston K, McManus S, Blanchard M, Erens B, et al. Common mental disorders and ethnicity in England: the EMPIRIC study. Psychol Med. 2004;34(8):1543-1551.
33. Mathur MB, Reichling DB. Navigating a social world with robot partners: A quantitative cartography of the Uncanny Valley. *Cognition*. 2016;146:22-32.
34. Lee YH, Park BN, Kim SH. The effects of heat and massage application on autonomic nervous system. *Yonsei Med J*. 2011;52(6):982-989.

For peer review only - http://bmjopen.bmj.com/site/about/guidelines.xhtml

35. Dillon KM, Seacat JD, Saucier CD, Doyle-Campbell CJ. Could Blood Pressure Phobia Go Beyond the White Coat Effect? *Am J Hypertens*. 2015;28(11):1306-1309.
36. Lazard AJ, Watkins I, Mackert MS, Xie B, Stephens KK, Shalev H. Design simplicity influences patient portal use: the role of aesthetic evaluations for technology acceptance. *J Am Med Inform Assoc*. 2016;23(1):157-161.
37. Stanley E, Cradock A, Bisset J, McEntee C, O'Connell MJ. Impact of sensory design interventions on image quality, patient anxiety and overall patient experience at MRI. *Br J Radiol.* 2016;89(1067).
38. Prakash B. Patient satisfaction. *J Cutan Aesthet Surg.* 2010;3(3):151-155.

For peer review only

Figure 1- An outline of the overarching themes and the subsequent subthemes within. The Venn diagram demonstrates where the patients and HCPs agree and disagree with each other.

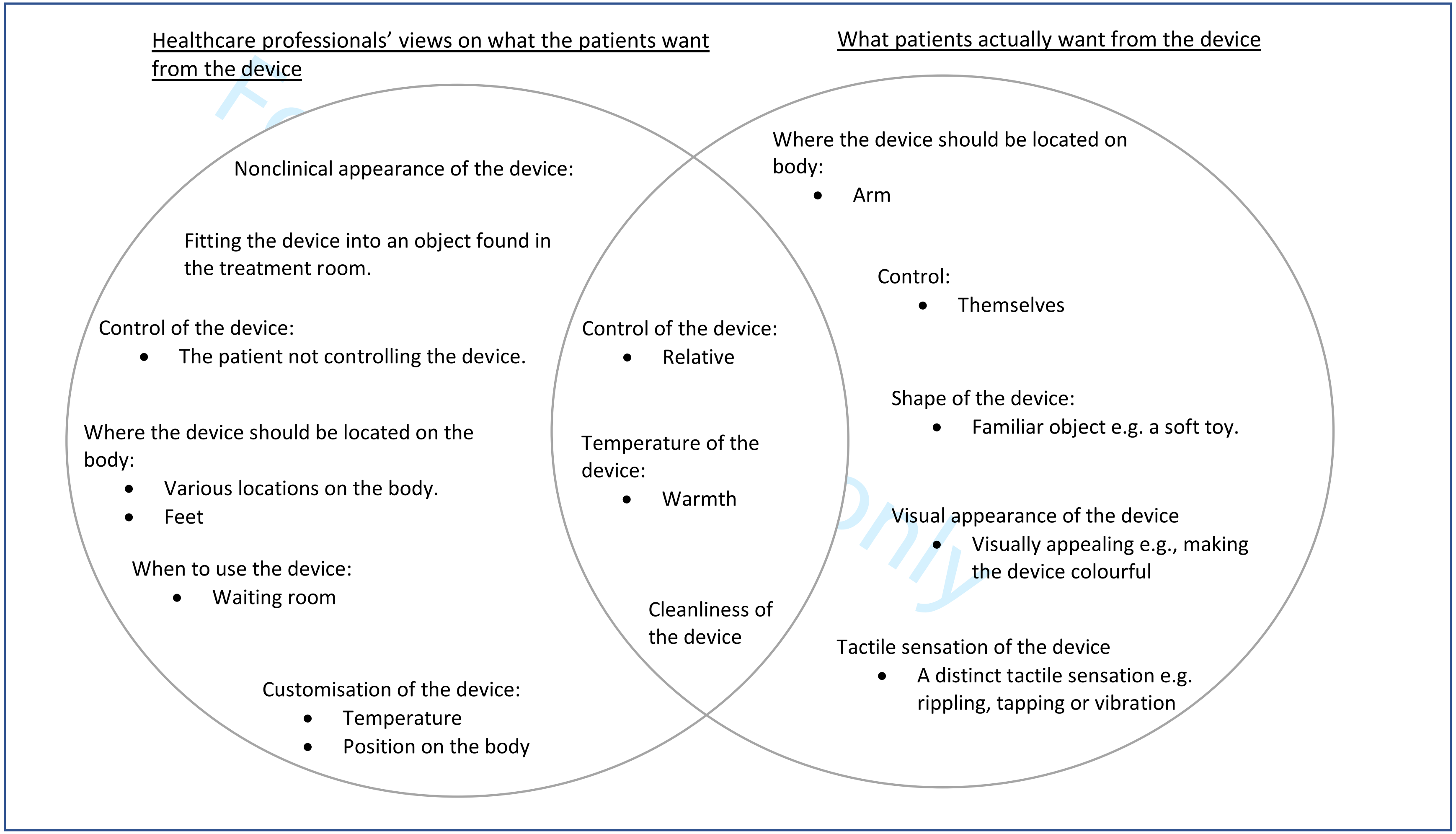

Standards for Reporting Qualitative Research (SRQR) as it applies to:

PATIENT AND PRACTITIONER PERSPECTIVES ON THE DESIGN OF A SIMULATED AFFECTIVE TOUCH DEVICE TO REDUCE PROCEDURAL ANXIETY ASSOCIATED WITH RADIOTHERAPY: A QUALITATIVE STUDY

O'Brien B.C., Harris, I.B., Beckman, T.J., Reed, D.A., & Cook, D.A. (2014). Standards for reporting qualitative research: a synthesis of recommendations. *Academic Medicine, 89(9)*, 1245-1251.

No. Topic	Location in Article (page number)
**Title and abstract**	N.B. Page numbers are based off the proof page numbers.
S1 Title	Page 2
S2 Abstract	Page 3
**Introduction**	
S3 Problem formulation	Page 5
S4 Purpose or research question	Page 6
**Methods**	
S5 Qualitative approach and research paradigm	Page 6
S6 Researcher characteristics and reflexivity	Page 8-9
S7 Context	Page 7
S8 Sampling strategy	Page 7
S9 Ethical issues pertaining to human subjects	Page 6
S10 Data collection methods	Page 7
S11 Data collection instruments and technologies	Page 7
S12 Units of study	Page 7-8
S13 Data processing	Pages 8
S14 Data analysis	Page 8-9
S15 Techniques to enhance trustworthiness	Page 8-9
**Results/Findings**	
S16 Synthesis and interpretation	Pages 9-13
S17 Links to empirical data	Pages 9-13
**Discussion**	

For peer review only

S18 Integration with prior work, implications, transferability, and contribution(s) to the field	Pages 13-15
S19 Limitations	Page 15
**Other**	
S20 Conflicts of interest	Page 2
S21 Funding	Page 2

For peer review only
